# A Natural Latex-Based Smart Dressing for Curcumin Delivery Combined with LED Phototherapy in Diabetic Foot Ulcers: A Pilot Clinical Study

**DOI:** 10.3390/pharmaceutics17060772

**Published:** 2025-06-12

**Authors:** Thamis Fernandes Santana Gomes, Natália Carvalho Guimarães, Ludmilla Pinto Guiotti Cintra Abreu, Gabriella de Oliveira Silva, Vitória Regina Pereira da Silva, Franciéle de Matos da Silva, Fabiane Hiratsuka Veiga-Souza, Paulo Eduardo Narcizo de Souza, Mário Fabrício Fleury Rosa, Graziella Anselmo Joanitti, Suélia de Siqueira Rodrigues Fleury Rosa, Marcella Lemos Brettas Carneiro

**Affiliations:** 1 Laboratory of Bioactive Compounds and Nanobiotechnology (LCBNano), University of Brasilia, Brasilia 70910-900, DF, Brazil; thamisunb@gmail.com (T.F.S.G.); ludguiotti2@gmail.com (L.P.G.C.A.); oliveiragaby101@gmail.com (G.d.O.S.); vitoriarpsilva@gmail.com (V.R.P.d.S.); gjoanitti@unb.br (G.A.J.); 2 Laboratory of Protein Chemistry and Biochemistry, Department of Cell Biology, Institute of Biology, University of Brasilia, Brasilia 70910-900, DF, Brazil; nataliacguimaraes@hotmail.com (N.C.G.); fhveiga@unb.br (F.H.V.-S.); 3 Postgraduate Program in Biomedical Engineering at Gama-FGA, University of Brasilia, Brasilia 70910-900, DF, Brazil; mariofleuryrosa@gmail.com (M.F.F.R.); sdf73@cornell.edu (S.d.S.R.F.R.); 4 Laboratory of Microscopy and Microanalysis, Department of Cell Biology, Institute of Biological Sciences, University of Brasilia, Brasilia 70910-900, DF, Brazil; francielematos75@gmail.com; 5 Laboratory of Experimental Biomodels (LaBiEx), University of Brasilia, Brasilia 70910-900, DF, Brazil; 6 Laboratory of Electron Paramagnetic Resonance, Institute of Physics, University of Brasilia, Brasilia 70910-900, DF, Brazil; psouza1974@gmail.com; 7 Postgraduate Program in Mechanical Sciences, University of Brasilia, Brasilia 70910-900, DF, Brazil; 8 Meinig School of Biomedical Engineering, Master of Engineering (M.Eng.) Program, Cornell University, Ithaca, NY 14853, USA

**Keywords:** curcumin, natural latex biomembrane, wound healing, ulcers, oxidative stress, clinical assay

## Abstract

**Background**: Diabetic foot ulcers (DFUs) affect 25% of diabetes patients, with high risks of amputation (70%), recurrence (65% within 3–5 years), and mortality (50–70% at 5-years). Current treatments are limited by persistent inflammation, oxidative stress, and cost barriers. This study evaluates a bioactive dressing combining a natural latex-based (NLB) biomembrane (*Hevea brasiliensis*) with curcumin-loaded liposomes, exhibiting angiogenic and antimicrobial properties, and red LED (light-emitting diode) phototherapy (635–640 nm) to address these challenges. **Methods**: A pilot clinical trial randomized 15 DFU participants into three groups: Control (CG, *n* = 5, standard care); Experimental Group 1 (EG1, *n* = 5, NLB + LED, daily treatment); and Experimental Group 2 (EG2, *n* = 5, NLB-curcumin liposomes + LED, daily treatment). Outcomes included wound closure, inflammatory/oxidative markers, and therapy feasibility. Assessments at D0, D22, and D45 included hematological/biochemical profiling, reactive oxygen species (ROS), and wound area measures. **Results**: On day 45, GE2 showed an average ulcer contraction of 89.8%, while CG showed 32.8%, and GE1 showed 9.7%. Systemic ROS and biomarkers (C-reactive protein, leukocytes) showed no significant changes (*p* > 0.05), though transient inflammatory spikes occurred. The combined therapy (EG2) accelerated healing without direct biomarker correlations. **Conclusions**: These findings highlight the potential of this combined therapy as an accessible, cost-effective DFU treatment, warranting larger studies to optimize home-based protocols and elucidate mechanisms.

## 1. Introduction

Diabetic foot ulcers (DFUs) are chronic wounds characterized by the disruption of the epidermis and at least part of the dermis in individuals with diabetes, commonly accompanied by neuropathy and/or peripheral arterial disease [1]. DFUs are frequent complications among diabetics, manifesting in around 25% of these individuals and carrying a 70% risk of amputation [2]. These ulcers also contribute to significant morbidity, with a high recurrence rate of 65% within 3 to 5 years and a 5-year mortality rate ranging from 50 to 70%. Pathogenesis involves DFU sensory loss, ischemia, and minor trauma, with ulcers classified as neuropathic (35%), ischemic (15%), and neuroischemic (50%). The Wound, Ischemia, and foot Infection (WIfI) system by the Society for Vascular Surgery is a validated tool for risk stratification and management [3].

Chronic diabetic wounds (>3 months duration) exhibited elevated inflammatory cytokines, matrix metalloproteinases (MMP), and reactive oxygen species (ROS). Hyperglycemia exacerbates impaired healing by disrupting keratinocyte interactions, damaging Schwann cells, and reducing microcirculation. Persistent ROS production accelerates advanced glycation end products (AGEs) formation, which promotes abnormal protein cross-linking, growth factor dysfunction, and nitric oxide impairment. AGE-receptor interactions further hinder tissue repair via collagen rigidity, reduced elasticity, and increased injury susceptibility [2,4].

In diabetic wounds, persistent infection disrupts the normal stages—hemostasis, inflammatory response, regeneration, and tissue remodeling. Keratinocytes exhibit impaired proliferation and migration, while macrophages and fibroblasts undergo senescence, perpetuating ROS release and chronic inflammation [2]. Current guidelines emphasize structured wound assessment and the TIME principle (tissue management, infection control, moisture balance, wound edge) for treatment. Moist dressings (e.g., hydrogels, amniotic membranes) are preferred over traditional gauze, accelerating healing by 40% compared to dry environments. However, traditional dressings like gauze and bandages often adhere to wounds and lack advanced therapeutic properties [5]. To address these limitations, novel approaches combining biomaterials, phytotherapy, nanotechnology, and phototherapy have emerged as promising strategies for enhancing wound healing in DFUs.

Biomaterials have set new standards in modern wound management, particularly those with bioactive properties capable of modulating inflammation and promoting tissue regeneration [6,7]. Natural, synthetic, and hybrid biomaterials are being increasingly engineered for wound healing applications, offering advantages such as biocompatibility, sustained drug release, and enhanced structural support [7]. Among biomaterials, natural latex-based (NLB) membranes derived from *Hevea brasiliensis* exhibit angiogenic, antimicrobial, and tissue-regenerative properties, making them promising for DFU management [8]. The natural latex is a polydisperse material composed of rubber particles encased in a thin layer of proteins, lipids, and minerals extracted from the *Hevea brasiliensis* tree [9,10]. NLB exhibits properties such as increased vascular permeability, antifungal and antimicrobial activities, angiogenic action, and wound healing capabilities [11,12,13,14].

In addition, the use of phytotherapeutic compounds in wound healing has gained significant attention due to their natural bioactive properties, which promote tissue regeneration with minimal side effects. Among these compounds, curcumin polyphenol derived from *Curcuma longa* stands out for its anti-inflammatory, antioxidant, and antimicrobial activities, which accelerate wound closure and enhance collagen deposition [15,16]. However, curcumin’s poor bioavailability limits its clinical application, prompting the development of nanostructured delivery systems such as liposomes [17,18,19,20].

These lipid-based carriers improve curcumin’s solubility, stability, and targeted delivery, enhancing its therapeutic potential in wound healing. Liposomes, due to their amphiphilic nature, can encapsulate both hydrophilic and hydrophobic compounds like curcumin, enhancing its penetration into damaged tissues while protecting it from degradation [21]. These vesicles also mimic biological membranes, reducing toxicity and improving biocompatibility. Studies demonstrate that curcumin-loaded liposomes significantly enhance fibroblast proliferation and angiogenesis, critical processes in tissue regeneration [22]. Thus, the combination of phytotherapeutics and nanotechnology, particularly liposomal formulations, represents a cutting-edge approach for improving wound healing therapies [23].

Otherwise, photobiomodulation (PBM), particularly LED phototherapy, uses red light (635–640 nm) to stimulate cellular repair mechanisms. LED therapy offers advantages such as cost-effectiveness, safety, and suitability for home use [24,25,26,27]. The absorption of light by chromophores leads to the production of adenosine triphosphate (ATP), triggering molecular, cellular, and tissue cascades that contribute to therapeutic effects [28]. Clinically, LED phototherapy has been shown to stimulate collagen production, activate fibroblasts, promote angiogenesis, reduce inflammation, and decrease wound area [24,25]. It also fosters tissue regeneration by reducing reactive oxygen species (ROS) levels, facilitating cellular proliferation, granulation tissue formation, and re-epithelialization [29].

In the context of diabetic foot ulcers, the Rapha^®^ phototherapy device has been developed. This device features a panel with 30 LEDs operating at wavelengths between 635 and 640 nm (red light) and is used in conjunction with NLB [30,31]. Clinical trials have demonstrated that this system accelerates wound healing and improves ulcer recovery compared to standard treatments [32]. The therapy promotes granulation tissue formation, reduces non-viable tissue, facilitates wound edge contraction and closure, and absorbs exudate while maintaining necessary moisture. Additionally, the ability to perform the treatment at home enhances the patient’s quality of life by reducing the need for frequent hospital visits [29,33]. Moreover, when combined with NLB membranes, LED phototherapy enhances wound closure and tissue regeneration, offering a practical solution for at-home care [31].

This study evaluated a novel bioactive wound dressing composed of a natural latex-based biomembrane with curcumin-loaded liposomes and LED phototherapy for DFU management. The primary objectives were to investigate its clinical efficacy in wound closure, inflammation reduction, and oxidative stress modulation, while secondary goals included safety, feasibility, and practicality for home use. By integrating these advanced therapies, this research seeks to provide a cost-effective, accessible, and scientifically validated treatment alternative for diabetic patients with chronic foot ulcers, ultimately improving healing outcomes and quality of life.

## 2. Materials and Methods

### 2.1. Materials

The phototherapy device used in this study was the Rapha^®^ system, manufactured by Easy Things (Brasilia, DF, Brazil). The equipment consists of a panel containing 30 high-power light-emitting diodes (LEDs), emitting red light within the 635–640 nm wavelength range. The system delivers a total power output of 1800 mW and an energy density of 25 J/cm^2^, applied through sequential pulsed light with a programmed irradiation time of 35 min per session.

The natural latex-based biomembranes were produced from prevulcanized, bicentrifuged natural latex extracted from the *Hevea brasiliensis* tree, supplied by Du Latex Ltd. (São Paulo, Brazil), following the standardized protocol established by Rosa et al. [31].

The curcumin used in the liposomal formulation was purchased from Sigma-Aldrich (St. Louis, MO, USA), with ≥95% purity (HPLC grade). Liposomes were formulated using L-α-phosphatidylcholine (from soybean, ≥99% purity), acquired from Avanti Polar Lipids (Alabaster, AL, USA), and cholesterol (≥99% purity, Sigma-Aldrich). All reagents used were of analytical or pharmaceutical grade. Curcumin-loaded liposomes were prepared using the lipid film hydration method [34], involving solvent evaporation and subsequent rehydration, as described by Silva [23]. The resulting liposomes were incorporated into the latex matrix before vulcanization to ensure homogeneous dispersion of the phytotherapeutic agent within the biomembrane.

The curcumin concentration applied in the biomembranes was empirically determined based on prior in vivo experiments in diabetic wound models, balancing therapeutic efficacy and membrane stability. The specific dose per unit area (µg/cm^2^) will be reported in a forthcoming study currently in preparation.

The liposomal vesicles had an average size of 905.5 ± 122.3 nm, a polydispersity index of 0.764, and a zeta potential of −40.7 ± 2.22 mV, indicating moderate colloidal stability. They were rounded, multilamellar vesicles, consistent with the expected structural characteristics of multilamellar liposomes. These features support the structural integrity and expected behavior of the liposomal formulation.

### 2.2. Methods

#### 2.2.1. Study Design

This controlled clinical trial enrolled fifteen participants with diabetic foot ulcers meeting outpatient treatment criteria. The study population included adults aged 18–75 years old, with type I or II diabetes mellitus presenting with lower limb ulcers persisting for at least three weeks. Key inclusion criteria were the absence of latex allergies and preserved mental autonomy. We excluded participants with irregular medication use, evidence of osteomyelitis or gangrene, regular consumption of alcoholic beverages or illicit drugs, debilitating chronic conditions, breastfeeding status, or recent participation in other clinical trials. Participants were allocated into three treatments with five individuals each. The control group (CG) received standard wound care according to Brazilian Unified Health System (BUHS) protocols, using calcium alginate, hydrogel, or silver-impregnated activated charcoal dressings as medically indicated.

Experimental Group 1 (EG1) was treated with a natural latex biomembrane combined with LED phototherapy using the Rapha^®^ device emitting light at 635–640 nm. Experimental Group 2 (EG2) received the same phototherapy protocol but with a modified natural latex biomembrane incorporating curcumin-loaded liposomes. The proposed interventions were applied in an outpatient or home environment, depending on the treatment used.

**Figure 1:**
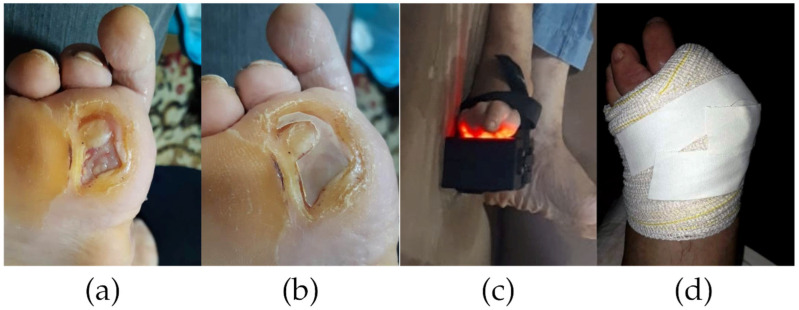
Wound treatment protocol using latex biomembrane and LED phototherapy. (**a**) Pretreatment presentation of the diabetic ulcer following aseptic cleansing with 0.9% saline solution. (**b**) Custom-sized natural latex biomembrane being applied to cover the wound bed. (**c**) Combined therapeutic intervention showing simultaneous biomembrane application and LED phototherapy (635–640 nm wavelength) administration. (**d**) Completed wound dressing with biomembrane as primary layer, secured by secondary gauze and bandage coverage [35]. Reproduced with permission from Thamis Fernandes Santana, Interdisciplinaridade no contexto das doenças dos pés no diabetes; published by EDUERN, 2021.

The control group (CG) received weekly professional wound care with instructions for home dressing changes as needed. All participants began treatment with wound cleansing using 0.9% saline solution (Figure 1a). For the experimental group (EG1 and EG2), this was followed by application of the appropriate biomembrane as primary dressing (Figure 1b) and LED irradiation for 35 min at an energy density of 25 J/cm^2^ (Rapha^®^ device, Figure 1c). The dressing was completed with sterile gauze and bandage, with the biomembrane remaining in place until the next scheduled change (Figure 1d). Experimental Group 1 (EG1) performed this protocol daily, while Experimental Group 2 (EG2) alternated between curcumin-loaded biomembrane and standard biomembrane (without curcumin-loaded liposome) every other day. In EG2, curcumin-loaded and non-loaded biomembranes were applied on alternating days due to logistical limitations in the production scale of the curcumin-loaded formulation at the time of the study.

All treatments continued for 45 days with regular monitoring. Home-based interventions were performed either independently by participants or with caregiver assistance after comprehensive training in the proper techniques. This study design allowed for comparison between standard care and two levels of experimental intervention while controlling treatment duration and monitoring frequency.

Longitudinal biological sampling was conducted at three key timepoints: baseline (day 0), interim (day 22), and study completion (day 45). All sample collections were performed immediately before scheduled dressing changes to ensure standardized timing relative to treatment administration. The comprehensive sampling protocol included venous blood draws for systemic analysis, wound tissue biopsies for local assessment, and standardized digital wound imaging for morphological evaluation.

This multimodal approach enabled concurrent evaluation of both systemic inflammatory markers through hematological analysis and local wound microenvironment changes through tissue examination. Blood samples were collected using sterile venipuncture technique and processed according to established protocols for serum separation and storage.

Wound tissue specimens were obtained under aseptic conditions using 3 mm punch biopsies from the wound edge, ensuring representative sampling of the healing interface. High-resolution photographic documentation was systematically performed using calibrated imaging equipment with fixed distance and lighting parameters to enable quantitative wound area analysis.

The Integrated sample collection strategy was designed to correlate biochemical markers of inflammation with clinical healing progression across all treatment groups while maintaining procedural consistency throughout the study duration. Particular attention was given to synchronizing sample collection with treatment timepoints to capture the temporal dynamics of therapeutic responses while minimizing confounding variables related to treatment timing.

#### 2.2.2. Clinical and Demographic Analysis

A rigorous demographic and clinical characterization was conducted for all study participants, encompassing both baseline characteristics and disease-specific parameters. Comprehensive demographic data collection included sex, age, height, marital status, and current employment status.

Clinical evaluation incorporated detailed medical history documentation with particular emphasis on diabetes duration and management, coexisting hypertension, and current mobility status. Lifestyle factors were systematically recorded, including smoking history and alcohol consumption patterns. Anthropometric measurements consisted of weight and body mass index (BMI) determinations, while metabolic control was assessed through both fasting blood glucose and glycated hemoglobin (HbA1c) levels.

Wound characterization followed standardized protocols using the University of Texas Diabetic Foot Ulcer Classification System [36], which enabled systematic documentation of ulcer type, depth, and associated pathological features. This multidimensional assessment framework ensured comprehensive participant profiling while maintaining consistency with established diabetic foot ulcer research methodologies.

All clinical measurements were performed by trained personnel using calibrated equipment following standardized operating procedures to ensure data reliability and reproducibility throughout the study duration.

#### 2.2.3. Hematological and Biochemical Analysis

Safety monitoring was conducted through comprehensive hematological and biochemical profiling. For these analyses, approximately 4 mL of venous blood was collected via standard phlebotomy techniques at each designated timepoint.

All laboratory processing was performed by the Research Support Center of the Sabin Institute following stringent quality control protocols. Complete blood count analysis was conducted using an automated XN-10 Sysmex hematology analyzer (Sysmex Brasil, São José dos Pinhais, Brazil), which simultaneously employs fluorescent flow cytometry for cellular differentiation and impedance technology for quantitative cellular enumeration. The biochemical panel incorporated multiple analytical methodologies standardized by Sabin Diagnostic Medicine Laboratory, including enzymatic colorimetry, immunoturbidimetry, and ion-selective electrode measurements, as detailed in Supplementary Table S1.

#### 2.2.4. Analysis of Wound Healing

Standardized wound documentation was performed through digital imaging at baseline (day 0), interim (day 22), and final (day 45) timepoints of the therapeutic protocol. All images were quantitatively analyzed using ImageJ software (version 1.54g, National Institutes of Health, Bethesda, MD, USA), employing a rigorous image processing pipeline to ensure measurement consistency.

The analytical workflow is initiated with image conversion to 8-bit grayscale format to optimize tissue contrast and segmentation efficiency. Spatial calibration was systematically performed using a 1 cm^2^ reference marker included in each photographic frame, with pixel-to-physical dimension conversion achieved through ImageJ’s set scale function to establish precise metric correlation.

Wound boundaries were delineated as regions of interest (ROI) using the polygon selection tool, with supplementary thresholding algorithms applied when necessary to enhance differentiation between viable and non-viable tissue. Following segmentation, wound areas were quantified in pixel units through the software’s measurement function and converted to absolute metric values (cm^2^) using the predetermined calibration factor.

Wound closure dynamics were calculated as percentage reduction relative to baseline according to Equation (1), where A₀ represents the initial wound area (day 0) and A₁ denotes the wound area at subsequent evaluations (days 22 and 45). The percentage of wound closure, Equation (1), over time was calculated by comparing the wound areas at each time point according to the established formula. This standardized analytical approach enabled objective, reproducible quantification of therapeutic efficacy while accounting for individual wound geometry variations throughout the treatment course.

Wound closure percentage (%) = (A_0_ − A_1_/A_0_) × 100 (1)

A_0_ = initial wound area on day 0

A_1_ = wound area on the day of measurement (days 22 and 45)

#### 2.2.5. Reactive Oxygen Species Quantification in Human Blood and Tissue

ROS quantification in blood and wound tissue samples was performed using electron paramagnetic resonance (EPR) spectroscopy with the spin probe CMH (1-hydroxy-3-methoxycarbonyl-2,2,5,5-tetramethylpyrrolidine), following an adapted protocol from López-Delis et al. [29]. All solutions were prepared fresh in Krebs HEPES buffer (KHB, pH 7.4), using bidistilled deionized water. The CMH stock solution (10 mM) was prepared in KHB containing 25 μM deferoxamine (metal chelator) and 5 μM sodium diethyldithiocarbamate trihydrate (DETC) to prevent probe oxidation.

For blood analysis, venous and wound blood samples were collected and immediately mixed 1:1 with a solution containing 400 μM CMH and 100 IU/mL sodium heparin. After 30-min incubation at room temperature, 50 μL aliquots were placed between two ice blocks (200 μL each) in 1 mL syringes and rapidly frozen in liquid nitrogen.

Wound tissue samples were collected and flash-frozen in liquid nitrogen, then processed by washing twice with 200 μL KHB buffer. Subsequently, 500 μL of a reaction solution containing 200 μM CMH and 50 IU/mL heparin was added to each tissue sample, followed by gentle shaking incubation at 37 °C for 1 h. The supernatant (450 μL) was then transferred to syringes and snap-frozen, while the remaining tissue was dried in a speed vacuum concentrator at room temperature and weighed using a precision analytical balance (AB135-S/FACT, Mettler Toledo, Columbus, OH, USA). All samples were stored at −80 °C until EPR analysis.

EPR measurements were conducted using an EMX Plus spectrometer (Bruker, Bremen, Germany) operating in X-band (9 GHz) with a high-sensitivity resonator (ER 4119HS). Frozen samples were analyzed at 77 K using a liquid nitrogen dewar (Noxygen, Elzach, Germany) with the following instrument parameters: 2 mW microwave power, 5 G modulation amplitude, 100 kHz modulation frequency, and 10 s sweep time. Spectra were acquired as the average of three sweeps across a 200 G field width. Quantification was achieved using a calibration curve generated with 3-carboxy-proxyl nitroxide radical (CP•) standards at concentrations of 0, 5, 10, 50, and 100 μM in KHB buffer. The method relies on the specific reaction of ROS with CMH to form the stable radical CM^•^ (3-methoxycarbonyl-2,2,5,5-tetramethylpyrrolidine-1-oxyl), whose EPR signal intensity directly correlates with ROS concentration in the sample. Representative EPR spectra for ROS quantification are provided in the Supplementary Information (Figure S1).

### 2.3. Statistical Analysis

All experimental data were processed using IBM SPSS Statistics (version 27, IBM Corp., Armonk, NY, USA) for statistical computations and GraphPad Prism (version 8.0, GraphPad Software) for graphical representations. Continuous variables are presented as mean ± standard deviation (SD) throughout the manuscript. Given the limited sample size inherent to this pilot clinical investigation (*n* = 5 per group), non-parametric statistical methods were employed for all analyses to ensure robustness against potential deviations from normality assumptions.

Intergroup comparisons among the three treatment arms were performed using the Kruskal–Wallis test. For significant Kruskal–Wallis results (*p* ≤ 0.05), Dunn’s post hoc test was conducted to identify specific differences between groups. Longitudinal changes within each treatment group at different time points (day 0 vs. day 22 vs. day 45) were assessed using the Friedman test for related samples, followed by Dunn’s post hoc test.

All statistical tests were two-tailed, with a α-level set at 0.05 for determination of statistical significance. This analytical approach provided appropriate statistical power while accounting for the small sample size and potential non-normal distribution of variables in this preliminary clinical investigation.

## 3. Results

### 3.1. Clinical and Demographic Analysis

Demographic analysis revealed that 80% of participants in each group—CG, EG1, and EG2—were male, consistent with the overall study population (Table S2). Regarding occupation, 47% of the cohort were employed, while 27% were retired, indicating that most participants had sufficient financial means to maintain hygiene and social engagement.

**Figure 2:**
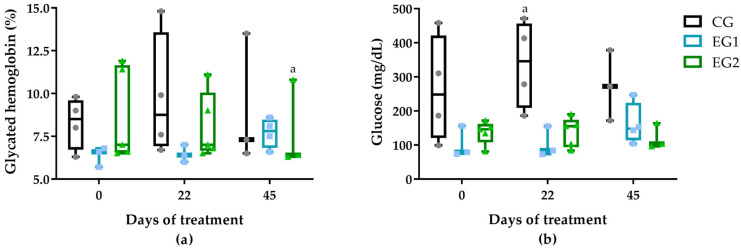
Glycated hemoglobin values (%) (**a**) and blood glucose concentration (**b**) among the treatment groups: standard therapy (CG), natural latex biomembrane with LED irradiation (EG1), and curcumin-loaded liposome biomembrane with LED irradiation (EG2) at days 0, 22, and 45. Data represent mean values ± standard deviation (*n* = 5 per group). Statistical significance (*p* ≤ 0.05) was determined by the Kruskal–Wallis test with post-hoc Dunn for more than two independent samples and Friedman test with post-hoc Dunn for related samples. Superscript letters indicate significant differences compared to day 0.

Clinically, 67% of the total population had hypertension, 60% used insulin, 33% were obese, 20% were mobility-dependent, 7% were smokers, and 33% consumed alcohol (Table S2). Comparative analysis of group characteristics demonstrated similar means for age, weight, height, BMI, HbA1c, and glucose (mg/dL) across all groups (Figure 2). However, the duration in years from the onset of diabetes mellitus (DM) and the duration in months of DFUs showed a lower average time and median in the group EG2, as seen in Table S3.

Wound classification analysis revealed that all ulcers in the CG were neuropathic, whereas the intervention groups (EG1 and EG2) displayed greater etiological variability (Table S4). In EG1, 60% of ulcers were neuropathic, 20% venous, and 20% neuroischemic. In EG2, 40% were neuropathic, 20% venous, 20% traumatic, and 20% neuroischemic. According to the University of Texas Classification, most ulcers (67%) were Grade 1 (superficial, without tendon, capsule, or bone involvement). However, the CG uniquely presented deeper wounds, with 60% Grade 1 and 40% Grade 3 (bone/joint involvement). In contrast, EG1 had 80% Grade 1 and 20% Grade 2 (tendon/capsule involvement), while EG2 had 60% Grade 1 and 40% Grade 2.

### 3.2. Hematological and Biochemical Analysis

**Figure 3:**
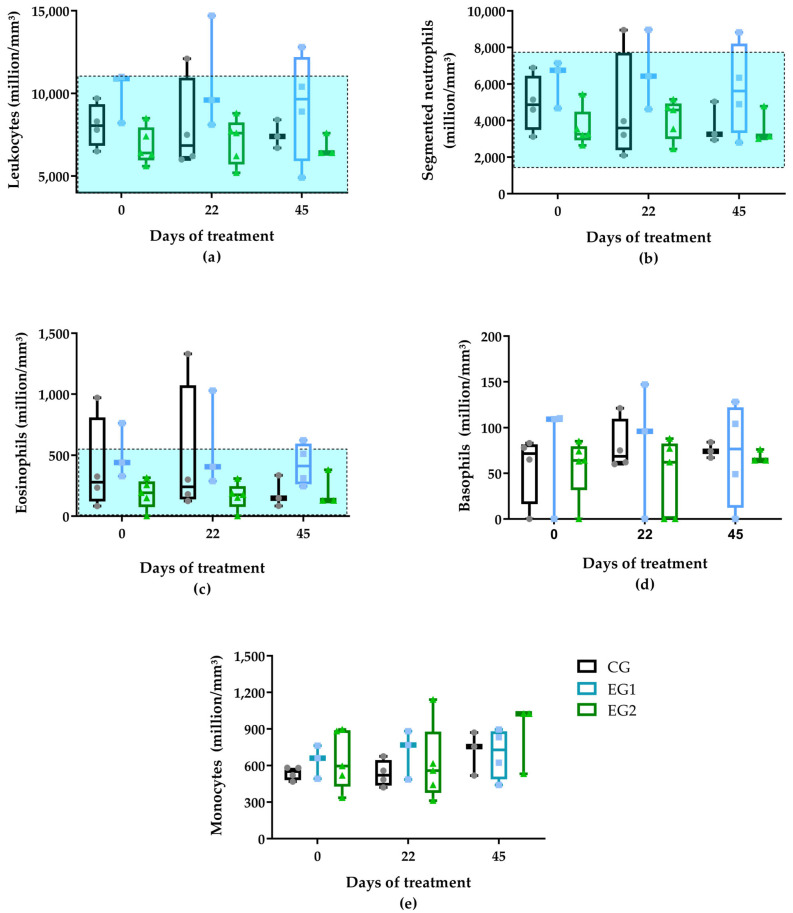
Leukogram cell counts: (**a**) leukocytes, (**b**) segmented neutrophils, (**c**) eosinophils, (**d**) basophils, and (**e**) monocytes among the treatment groups: standard therapy (CG), natural latex biomembrane with LED irradiation (EG1), and curcumin-loaded liposome biomembrane with LED irradiation (EG2) at days 0, 22, and 45. Data represent mean values ± standard deviation (*n* = 5 per group). Statistical significance (*p* ≤ 0.05) was determined by the Kruskal–Wallis test with post-hoc Dunn for more than two independent samples and Friedman test with post-hoc Dunn for related samples. The area of the rectangle represents the reference range.

Hematological and biochemical analyses were performed at baseline (D0), interim (D22), and post-treatment (D45) to assess participants’ health status. Initial comparisons (D0) showed no significant differences between groups for red blood cell (million/mm^3^), hemoglobin (g/dL), hematocrit (%), mean corpuscular volume (MCV) (fl), mean corpuscular hemoglobin (MCH) (pg), mean corpuscular hemoglobin concentration (MCHC) (g/dL), red cell distribution width (RDW) (%), lymphocytes (mm^3^), monocytes (mm^3^), platelets (×10^3^/mm^3^), and mean platelet volume (MPV) (fl). However, EG2 exhibited lower mean and median values for leukocytes, segmented leukocytes, eosinophils, and basophils compared to CG and EG1 at baseline (Figure 3).

In the control group (CG), leukocytes, segmented leukocytes, and eosinophils decreased between D0 and D45, while monocytes increased, though these changes were not statistically significant (Figure 3). The EG1 group showed transient elevations in leukocytes, segmented leukocytes, and eosinophils at D22 that returned to baseline by D45. The EG2 group maintained stable hematological parameters throughout, with only minor fluctuations in leukocyte subsets and monocytes observed at different timepoints (Tables S6–S8). However, there were no statistically significant changes.

**Figure 4:**
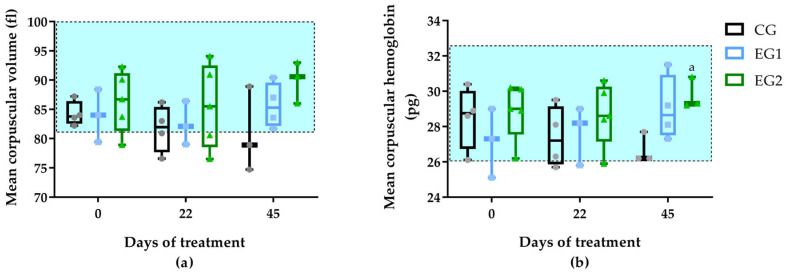
Mean corpuscular volume (**a**) and mean corpuscular hemoglobin (**b**) values among the treatment groups: standard therapy (CG), natural latex biomembrane with LED irradiation (EG1), and curcumin-loaded liposome biomembrane with LED irradiation (EG2) at days 0, 22, and 45. Data represent mean values ± standard deviation (*n* = 5 per group). Statistical significance (*p* ≤ 0.05) was determined by the Kruskal–Wallis test with post-hoc Dunn for more than two independent samples and Friedman test with post-hoc Dunn for related samples. Superscript letters indicate significant differences compared to the control group. The area of the rectangle represents the reference range.

Elevated MCV and MCH values were observed in all groups throughout the treatment period. However, only EG2 showed significantly higher MCH values compared to CG on D45 (*p* = 0.048) (Figure 4).

**Figure 5:**
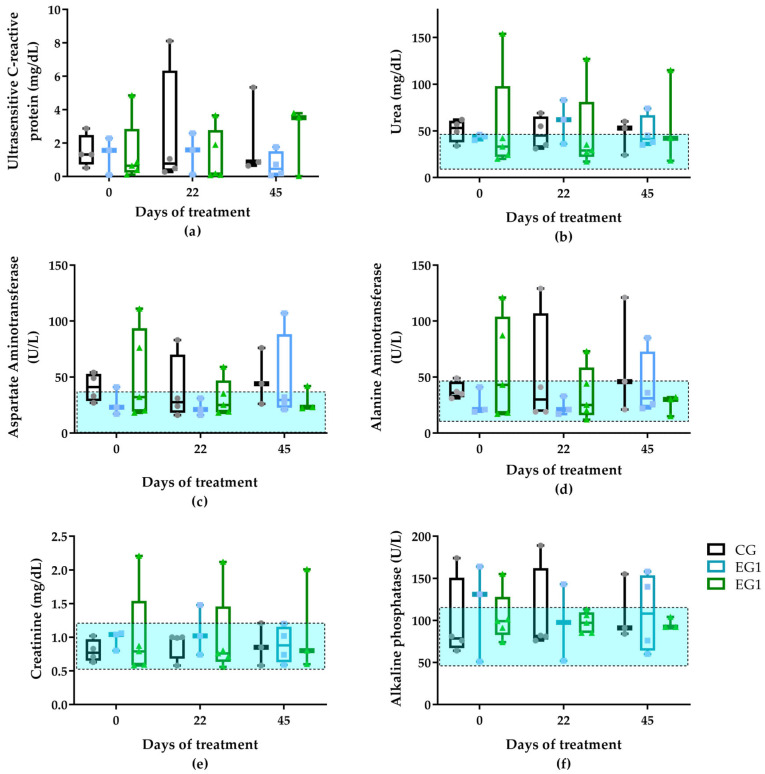
Concentrations of (**a**) ultrasensitive C-reactive protein, (**b**) urea, (**c**) aspartate aminotransferase, (**d**) alanine aminotransferase, (**e**) creatinine, and (**f**) alkaline phosphatase of the participants among the experimental groups: standard therapy (CG), natural latex biomembrane with LED irradiation (EG1), and curcumin-loaded liposome biomembrane with LED irradiation (EG2) at days 0, 22, and 45. Data represent mean values ± standard deviation (*n* = 5 per group). Statistical significance (*p* ≤ 0.05) was determined by the Kruskal–Wallis test with post-hoc Dunn for more than two independent samples and Friedman test with post-hoc Dunn for related samples. The area of the rectangle represents the reference range.

Biochemical analysis revealed that none of the participants between groups at D0 showed significant changes in their test results throughout the treatment (Table S9). Regardless of the CG had elevated baseline values for glucose (mg/dL), CRP (ultrasensitive C-reactive protein—67% above reference), urea (1% above reference), and AST (Aspartate Aminotransferase—17% above reference) (Figure 5).

The CG showed progressive increases in ALT from D0 to D45, along with minor rises in AST and total bilirubin, though these changes lacked statistical significance (Table S10). In EG1, urea, creatinine, and CRP-ultrasensitive peaked at D22, while AST, ALT, and alkaline phosphatase reached their highest levels at D45 (Figure 5). Similarly, EG2 demonstrated increased CRP-ultrasensitive and urea at D45, accompanied by a slight reduction in AST compared to baseline (Table S11).

Notable statistical differences included MCH values between EG2 and CG at D45 (*p* = 0.048), though both remained within normal ranges, and lower eosinophil counts in EG2 compared to EG1 (*p* = 0.025) (Table S12). All biochemical parameters were interpreted using established reference values [37].

### 3.3. Wound Healing Analysis

**Figure 6:**
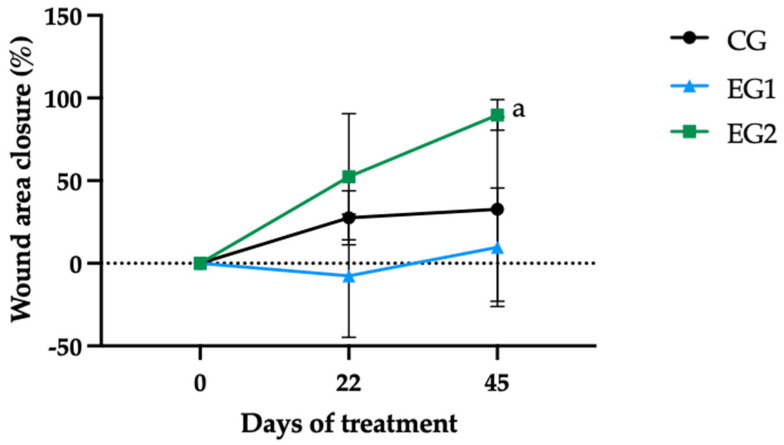
Temporal progression of wound closure (%) across experimental groups: standard therapy (CG), natural latex biomembrane with LED irradiation (EG1), and curcumin-loaded liposome biomembrane with LED irradiation (EG2) at days 0, 22, and 45. Data represent mean values ± standard deviation (*n* = 5 per group). Statistical significance (*p* ≤ 0.05) was determined by the Kruskal–Wallis test with post-hoc Dunn for more than two independent samples and Friedman test with post-hoc Dunn for related samples. Superscript letters indicate significant differences compared to day 0. The dashed reference line indicates baseline wound size (0% closure).

Wound healing progression was quantitatively assessed through serial measurements of wound area (cm^2^) at three time points: baseline (day 0), interim evaluation (day 22), and study conclusion (day 45). Percentage of healing was calculated using the standard Formula (1). Quantitative analysis revealed distinct healing trajectories among treatment groups. The experimental group receiving combined therapy (EG2) demonstrated superior healing outcomes, achieving 89.8% mean wound closure by day 45 (Figure 6).

Notably, group EG1 exhibited a transient 7.5% mean increase in wound area at day 22, though this variation lacked statistical significance compared to the control group (CG; *p* = 0.45). Comparative assessment revealed markedly different healing rates during the initial treatment phase, with EG2 achieving 52.4% closure versus CG’s 27.6% during the first 22 days (Figure 6).

By the end of the study, only the GE2 group exhibited a statistically significant progression in wound healing between days 0 and 45 (*p* = 0.013), indicating not only a faster but also a sustained therapeutic response. These findings highlight the potential of incorporating curcumin into the dressing as an effective strategy to enhance wound healing.

**Figure 7:**
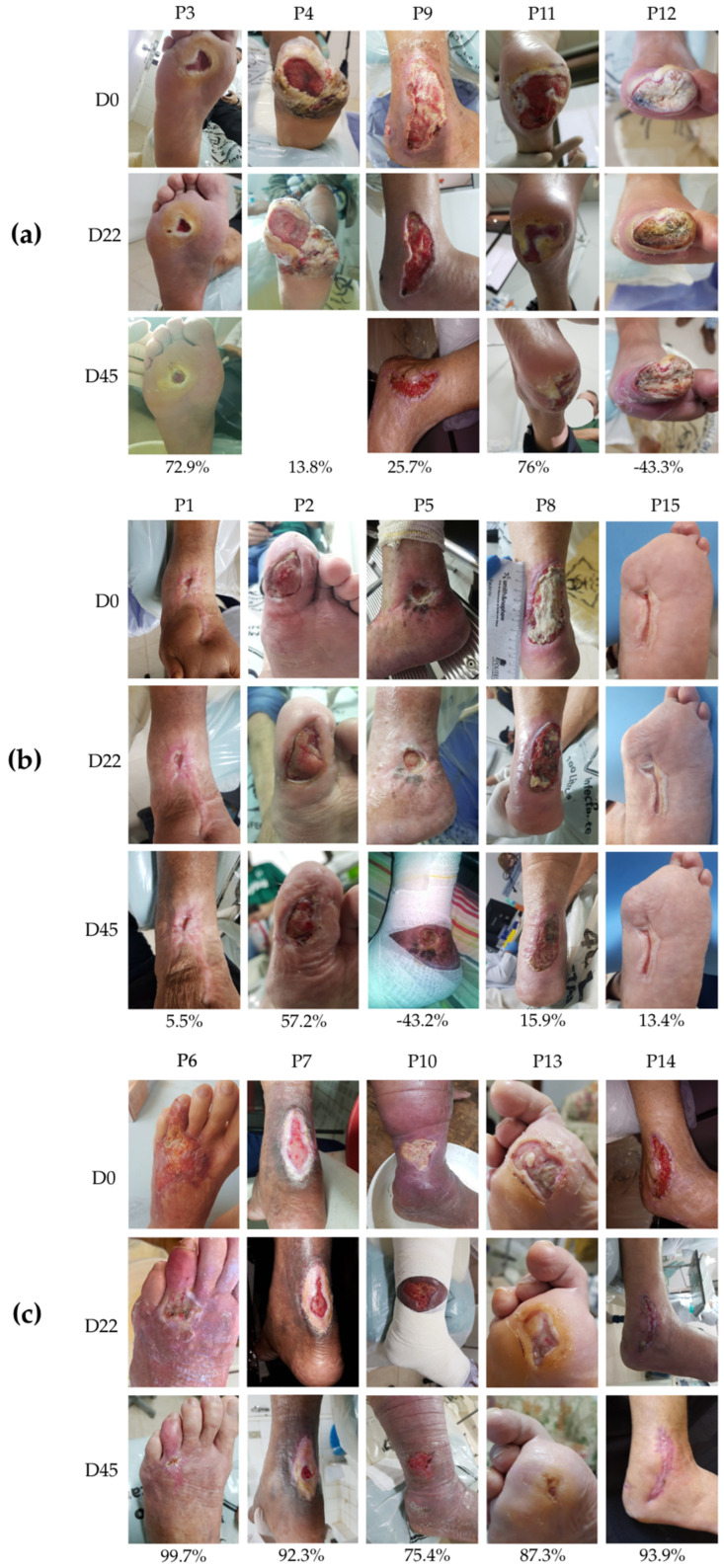
Representative wound images and corresponding wound closure percentages (WCP) at baseline (D0), interim (D22), and final evaluation (D45) for all participants across experimental groups: control group (CG, (**a**)), experimental group 1 (EG1, (**b**)), and experimental group 2 (EG2, (**c**)).

The photographic documentation of ulcer progression revealed considerable variability in wound closure percentage (WCP) among study participants (Figure 7). Patients treated with the BUHS protocol exhibited divergent healing outcomes: P3 and P11 demonstrated substantial wound area reductions of 72.9% and 76.0%, respectively (Figure 7a). In contrast, P9 achieved only a 25.7% reduction, while P12 showed no improvement, instead displaying a 43.3% increase in ulcer area. P4, who missed the final evaluation (D45), demonstrated a 13.8% healing rate during the observed period (D0–D22).

Photographic analysis revealed notable variability in wound healing responses across treatment groups. In EG1, participant P5 exhibited a 43% increase in wound area (Figure 7b), while P1 showed minimal improvement (5.5% closure). Other EG1 participants demonstrated intermediate healing rates: P2 (57.2%), P8 (15.9%), and P15 (13.4%).

The EG2 group, receiving curcumin-enhanced therapy, showed markedly superior outcomes. Three participants achieved >90% wound closure: P6 (99.7%), P14 (93.9%), and P7 (92.3%). The remaining EG2 participants still showed substantial improvement, with P13 at 87.2% and P10 at 75.4% closure (Figure 7c). These results demonstrate the significant therapeutic benefit of curcumin incorporation in the treatment protocol.

### 3.4. ROS Quantification in Blood and Tissues

**Figure 8:**
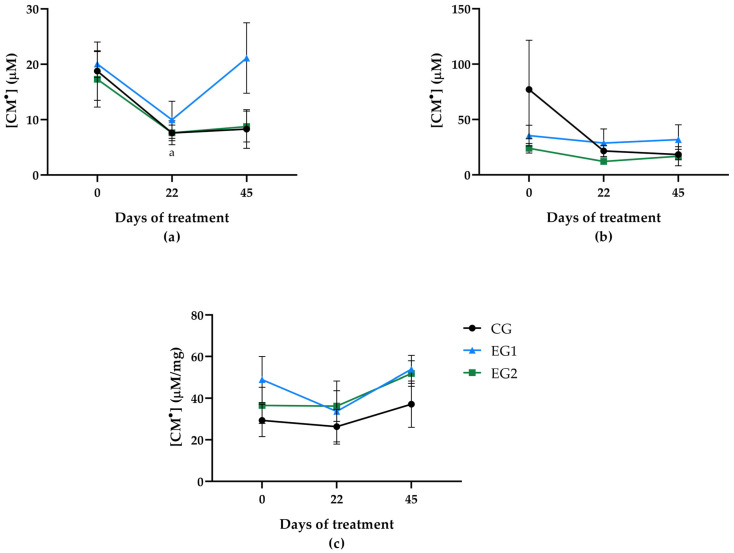
Reactive oxygen species (CM•) concentrations in (**a**) venous blood, (**b**) wound blood, and (**c**) wound tissue across treatment groups: standard therapy (CG), natural latex biomembrane with LED irradiation (EG1), and curcumin-loaded liposome biomembrane with LED irradiation (EG2) at days 0, 22, and 45. Data represent mean ± SEM (standard error of mean). Statistical significance (*p* ≤ 0.05) was determined by the Kruskal–Wallis test with post-hoc Dunn for more than two independent samples and Friedman test with post-hoc Dunn for related samples. Superscript letters indicate significant differences between days 0 and 22 in EG2.

Oxidative stress assessment through CM• quantification revealed distinct temporal patterns across treatment groups and biological samples. In venous blood, while no statistically significant intergroup differences emerged, both CG and EG2 demonstrated progressive CM• concentration decreases throughout treatment, with EG2 showing a statistically significant reduction between baseline and day 22. EG1 showed a biphasic response, with an initial reduction followed by terminal increase at D45 (Figure 8a).

Wound blood analysis demonstrated no significant intra-group temporal variations. However, CG displayed consistent CM• reduction from D0 to D45, EG1 maintained stable concentrations, and EG2 presented a characteristic pattern of mid-treatment reduction followed by slight terminal elevation (Figure 8b).

Wound tissue analysis revealed compartment-specific responses. CG showed progressive ROS accumulation, while both experimental groups exhibited terminal CM• increases at D45. EG1 demonstrated initial reduction followed by rebound, whereas EG2 maintained stable levels through D22 before the final increase (Figure 8c). These differential responses suggest treatment-specific modulation of oxidative stress across biological compartments.

## 4. Discussion

The demographic analysis (Tables S2 and S3) revealed a predominance of male patients (80%) with a median age of 59 years among DFU cases in our study, consistent with epidemiological patterns reported by the Brazilian Society of Angiology and Vascular Surgery (SBACV) [38] and supported by multiple international studies [39,40]. This gender disparity is particularly noteworthy, reporting a 2.2-fold higher ulceration risk in males, potentially attributable to occupational factors involving heavy physical labor. The age distribution in our cohort closely aligns with established literature documenting peak DFU incidence in patients aged ≥60 years [41].

Our occupational analysis revealed a distinct employment profile, with 47% of participants currently employed, compared to 27% retired and 27% unemployed. This distribution contrasts with previous clinical studies reporting higher proportions of retired individuals with limited education and incomes at or below minimum wage. The socioeconomic differences between our cohort and these reference populations may have important clinical implications, as lower educational attainment has been associated with reduced comprehension of self-care protocols, while financial constraints can negatively impact wound healing through multiple pathways: compromised nutrition, suboptimal hygiene conditions, and inadequate housing. These demographic distinctions should be considered when interpreting treatment outcomes and generalizing results to broader DFU populations [42,43].

The anthropometric analysis revealed that obesity (BMI ≥ 30) was present in only 33% of participants, with equal distribution across study groups. This finding contrasts with the established association between elevated BMI and poorer DFU outcomes, including impaired wound healing, chronic inflammation, and compromised immune function [44,45]. Notably, our cohort exhibited a high prevalence of hypertension (67%) and chronic wounds, characteristics that align with the global DFU profile described by Zhang et al. [40] in their systematic review. This international study identified typical DFU patients as elderly individuals with prolonged diabetes duration, hypertension, diabetic retinopathy, and smoking history—a profile that largely matches our study population except for the lower obesity prevalence. The dissociation between BMI and other metabolic risk factors in our sample warrants further investigation into potential protective mechanisms or population-specific characteristics influencing DFU progression [45].

Although lifestyle factors and comorbidities were documented during baseline assessment, the study design and sample size did not allow for statistical control of these variables. As such, residual confounding cannot be ruled out and may have influenced healing responses.

Notably, two participants in EG2 (P7 and P10) demonstrated significant wound healing improvement despite reported smoking and alcohol consumption. This observation appears contradictory to established evidence demonstrating the detrimental effects of these habits on tissue regeneration [46]. The apparent discrepancy may reflect individual variations in healing capacity or the potentially overriding therapeutic benefits of the curcumin-enhanced intervention in EG2. These findings should be interpreted considering the 2023 Brazilian Diabetes Society (SBD) guidelines, which emphasize that comprehensive metabolic control—including blood pressure management, lipid profile optimization, weight control, physical activity, and avoidance of smoking and excessive alcohol consumption—can significantly delay or prevent progression of diabetic complications [47]. The current results suggest that while lifestyle modifications remain crucial for optimal outcomes, advanced therapeutic interventions may partially mitigate the negative impact of certain risk factors in wound healing.

The metabolic parameters observed in our study population confirmed poor glycemic control across all participants, with mean HbA1c (7.9% ± 2.02) and glucose levels (170 ± 102.35 mg/dL) (Table S3) substantially exceeding diagnostic thresholds for diabetes mellitus (HbA1c ≥ 6.5%, fasting glucose ≥ 126 mg/dL), as established by the International Expert Committee and reaffirmed in the 2024 Brazilian Diabetes Society guidelines [48]. Notably, the control group (CG) demonstrated the poorest glycemic control throughout the study period, particularly from D22 onward (Figure 2), corresponding with the lowest wound contraction rate (27.6% between D0-D22, Table S6 and Figure 6).

In contrast, despite having the highest baseline HbA1c (8.68% ± 2.7), EG2 achieved superior therapeutic outcomes (52.4% wound contraction during the same period) following intervention with the curcumin-incorporated NLB combined with LED phototherapy (Table S3 and Figure 6). This apparent paradox suggests that while chronic hyperglycemia remains a critical factor in DFU pathogenesis, advanced topical therapies may partially overcome the negative metabolic milieu to promote wound healing. The findings align with current diagnostic standards, demonstrating 47–67% sensitivity and 98–99% specificity for HbA1c in diabetes diagnosis, while highlighting the need for comprehensive treatment approaches addressing both systemic metabolic control and localized wound management [48].

Current literature supports these laboratory parameters for diabetes diagnosis [49,50], with emerging evidence suggesting a non-linear relationship between glycemic control and wound healing. Notably, Xiang et al. [51] reported optimal healing rates at intermediate HbA1c levels (7.0–8.0%), with poorer outcomes observed at both lower (<7%) and higher (>8%) values. The detrimental effects of chronic hyperglycemia are well-documented, including impaired immune function, delayed tissue repair, enhanced bacterial proliferation, and increased biofilm formation—all contributing to antibiotic resistance [45].

Despite these established mechanisms, surprisingly few studies have systematically evaluated healing rates as a primary outcome measure of glycemic control [52], highlighting an important gap in current diabetes wound management research. Our findings contribute to this underexplored area by demonstrating significant wound improvement in EG2 despite elevated baseline HbA1c, suggesting that advanced topical therapies may help overcome some metabolic limitations to healing.

The study population exhibited a mean diabetes duration of 12 ± 7.7 years, consistent with established associations between disease chronicity and wound persistence (Table S3). Group-level analysis revealed clinically meaningful variations: EG2 had a lower average duration of DM (9 ± 7.64 years) and better wound healing results. EG1, on the other hand, had a longer DM duration (15 ± 6.53) and better results than CG (mean DM duration of 12 ± 9.09 years). These findings collectively demonstrate the enhanced efficacy of Rapha^®^ therapy compared to conventional BUHS treatment. The observed ulcer chronicity (mean duration 21 ± 31.64 months) further reinforces the challenging nature of these wounds, aligning with literature documenting poorer healing outcomes in long-standing diabetes (>10 years) [39].

Notably, the extended duration and larger size of DFUs in our cohort likely reflect greater disease severity, with such chronic wounds known to harbor complex microbial ecosystems that complicate treatment and promote antibiotic resistance [45]. The differential therapeutic responses observed across groups suggest that advanced therapeutics interventions like Rapha^®^ may partially mitigate the negative prognostic factors typically associated with prolonged diabetes duration and wound chronicity.

Hematological and biochemical analyses were conducted to assess potential systemic responses to DFU treatments. Baseline complete blood count parameters (D0) fell within normal reference ranges across all groups (Table S5). Although it showed elevated mean corpuscular hemoglobin (MCH) values compared to the control group (CG) on D45 (*p* = 0.048) (Figure 4), experimental group 2 (EG2) demonstrated higher ulcer contraction rates. This finding appears paradoxical given established literature indicating that: (1) elevated MCV values impair DFU healing by compromising microcirculation [53] and (2) both MCV and MCH increase with chronic hyperglycemia [54]. The dissociation between hematological parameters and clinical outcomes in our study suggests that the therapeutic benefits of the EG2 intervention (curcumin-incorporated biomembrane with phototherapy) may operate through mechanisms independent of erythrocyte indices, potentially involving localized anti-inflammatory and angiogenic effects that overcome systemic hematological constraints on wound healing.

Leukocyte analysis revealed consistent counts across all groups and timepoints (Tables S6–S8), except EG1, which showed transient leukocytosis at D22. This finding contrasts with established wound healing paradigms where leukocyte migration from circulation to wound sites typically produces measurable decreases in peripheral counts [55]. The expected inverse relationship between wound contraction (most pronounced in EG2) and circulating leukocytes was not observed, suggesting either: (1) sufficient bone marrow compensation maintained circulating pools despite tissue migration or (2) our sampling timeframe missed transient decreases. These results should be interpreted considering leukocytes’ dual roles as both inflammatory mediators [56] and microbial scavengers [57] during debridement phases. The dissociation between excellent clinical outcomes in EG2 and stable leukocyte counts implies that the curcumin-LED intervention may have enhanced healing through mechanisms other than gross leukocyte trafficking modulation, potentially via localized anti-inflammatory effects or improved microbial clearance efficiency. This improvement highlights the potential of targeted therapies to bypass traditional inflammatory pathways while still achieving optimal tissue repair.

While we monitored venous eosinophil levels throughout treatment, no direct correlation with wound healing outcomes emerged. Current evidence establishes eosinophils as multifunctional immune cells involved in: (1) glucose metabolism regulation, (2) tissue repair mechanisms, (3) epithelial differentiation [58], and (4) antimicrobial defense against viral, fungal, and bacterial pathogens [59]. During physiological wound healing, eosinophils typically migrate to wound sites during the proliferative phase, where they promote tissue repair through growth factor secretion. These mediators stimulate fibroblast proliferation and migration, facilitating tissue remodeling [60]. However, the systemic eosinophil profile during wound healing remains poorly characterized, as most studies focus exclusively on local wound infiltration rather than peripheral blood dynamics.

Of clinical relevance, participant P8 (EG1) exhibited marked eosinophilia (1029 cells/mm^3^) at D22, coinciding with elevated renal markers (urea: 83 mg/dL; creatinine: 1.48 mg/dL; Table S11). This hematological finding likely reflects concurrent renal impairment, as peripheral eosinophilia has been specifically associated with diabetic nephropathy pathogenesis [61]. The temporal association between deteriorating renal function and eosinophil elevation suggests either: (1) reduced renal clearance of eosinophils or (2) active eosinophil involvement in nephropathic inflammation. Therefore, the eosinophilia observed in this participant on D22 may be related to a possible kidney problem. This case underscores the importance of comprehensive metabolic monitoring during DFU treatment, particularly in patients with preexisting renal compromise.

The elevated urea levels observed across all groups at baseline (Tables S9–S11) suggest potential renal or hepatic impairment, though comprehensive comorbidity data were unavailable for confirmation. The control group (CG) demonstrated urea and AST levels exceeding reference values by 17% and 8%, respectively, during the D0–D22 period. Similarly, Experimental Group 2 (EG2) showed elevated urea (10% above reference), AST (34%), and ALT (14%) at D0, while Experimental Group 1 (EG1) exhibited urea and creatinine levels of 22% and 12% above reference at D22. These biochemical abnormalities are consistent with known diabetes complications, as chronic hyperglycemia induces both nephropathy and hepatopathy through multiple pathogenic pathways, including advanced glycation end-product accumulation and oxidative stress [62,63].

However, it is noteworthy that while hepatic and renal dysfunction frequently complicate diabetes management, a retrospective analysis by Rhou et al. [64] demonstrated that elevated AST and ALT levels do not necessarily correlate with impaired wound healing outcomes. This important finding suggests that metabolic derangements, while clinically significant for overall diabetes care, may not directly determine ulcer healing trajectories when addressed through targeted therapeutic interventions. The dissociation between biochemical markers of organ dysfunction and wound healing outcomes underscores the potential value of localized treatment approaches for diabetic foot ulcers that can achieve tissue repair despite systemic metabolic challenges.

The persistent elevation of urea levels across all study groups at baseline suggests underlying renal or hepatic impairment, though comprehensive comorbidity data were unavailable for definitive assessment. Notably, while these metabolic abnormalities are commonly associated with diabetes-related complications [62,63], their clinical significance for wound healing remains uncertain. A retrospective analysis by Rhou et al. [64] demonstrated that elevated AST levels showed no significant correlation with impaired ulcer healing outcomes, indicating that hepatic dysfunction markers may not reliably predict wound progression in diabetic patients. This important distinction suggests that while systemic metabolic derangements require clinical attention, they may not directly determine local tissue repair processes when managed through targeted therapeutic interventions.

Elevated C-reactive protein (CRP) levels persisted across all experimental groups throughout the treatment period (Tables S10–S12), contrary to the expected decline given CRP’s established association with reduced inflammation during wound healing [65]. Notably, only participants P7 and P10 in EG2 exhibited CRP levels exceeding reference values at D45 (87% and 86% above normal, respectively), corresponding with their comparatively poorer healing outcomes (87% and 66% wound closure, respectively). This correlation may reflect the persistent alcohol consumption and tobacco use reported by these individuals, as both substances are known to sustain systemic inflammation [66]. CRP serves as a sensitive biomarker for various inflammatory conditions, including diabetes, obesity, and cardiovascular disease, while elevated levels of CRP and erythrocyte sedimentation collectively indicate potential infection [45].

The observed variability in CRP profiles likely stems from individual patient characteristics within this small cohort (*n* = 5 per group). While these fluctuations may obscure group-level trends, they highlight important considerations for clinical interpretation: (1) lifestyle factors significantly influence inflammatory markers independent of treatment effects and (2) the therapeutic response to wound interventions may be modulated by baseline inflammatory status. Larger sample sizes would help distinguish between treatment effects and individual variability, potentially revealing clearer patterns of inflammatory marker dynamics during wound healing.

The pleiotropic biological effects of curcumin have been extensively documented, with demonstrated efficacy across multiple pathological conditions including cancer, diabetes, inflammatory diseases, and tissue repair processes [67]. At the molecular level, curcumin exerts its therapeutic effects through modulation of key genes involved in inflammatory regulation, epithelial proliferation, and angiogenesis—all critical pathways in wound healing, particularly in diabetic ulcer models [68]. These mechanisms align with recent findings by Cao et al. [67], who identified curcumin’s ability to downregulate miR-152-3p, a microRNA implicated in DFU pathogenesis through its negative effects on fibroblast proliferation, apoptosis induction, and suppression of fibrillin-1 (FBN-1) expression in both in vivo and in vitro hyperglycemic conditions.

Our clinical results substantiate these preclinical observations, with the curcumin-treated group (EG2) achieving 89.8% mean wound closure within 45 days. This superior performance compared to the non-curcumin intervention (EG1) suggests a synergistic effect between the liposomal curcumin formulation and phototherapy, potentially through enhanced bioavailability and targeted delivery [69,70]. The efficacy of our approach is further supported by preclinical studies reporting 86.6–100% wound closure within 9–12 days using various curcumin-incorporated dressings [71], though direct comparisons are limited by differences in experimental models and delivery systems. However, the experimental design of EG2, which involved alternating between biomembranes with and without curcumin, prevents the isolation of curcumin’s exclusive effect, unlike EG1, which received continuous treatment. Future studies must include groups with continuous use to more accurately assess the impact of curcumin.

While the formulation was designed for controlled curcumin release, this characteristic was not directly assessed in the present study. Future in vitro and in vivo investigations are required to characterize the release kinetics and degradation behavior of the liposomal biomembrane system under physiological conditions.

While intergroup comparisons did not reach statistical significance in our pilot study, the clinically meaningful trends observed warrant further investigation. Future controlled trials should incorporate: (1) larger sample sizes to enhance statistical power, (2) participant stratification by key risk factors, and (3) comprehensive biomarker profiling to identify predictors of therapeutic response. Such approaches may facilitate the development of personalized treatment strategies for complex diabetic wounds, optimizing the translational potential of curcumin-based therapies.

Our study evaluated oxidative stress by measuring reactive oxygen species (ROS) concentrations using CM^•^ detection in venous blood, wound blood, and wound tissue at baseline (D0), interim (D22), and final evaluation (D45). We found no significant intergroup differences in ROS levels during the treatment period, although EG2 demonstrated a statistically significant reduction in CM• concentrations in venous blood between baseline and day 22, indicating that latex curcumin plus LED combination (EG2) produced the most consistent antioxidant effect during the initial treatment phase. Despite this temporal reduction in EG2 and the improved wound healing observed in both EG1 and EG2, the overall pattern suggests that therapeutic benefits may occur primarily through mechanisms independent of sustained systemic oxidative stress modulation. The inflammatory phase of wound healing normally involves substantial ROS production by macrophages and neutrophils, which play critical roles in pathogen defense [72,73]. However, excessive ROS generation leads to oxidative stress—an imbalance between oxidants and antioxidants that damages cellular components and delays healing [74]. This pathological state is particularly relevant in chronic wounds, where persistent oxidative stress contributes to impaired tissue repair [75].

Our findings demonstrate a distinct temporal pattern of ROS dynamics compared to the study by López-Delis et al. [29], who reported elevated wound ROS levels at baseline, followed by a biphasic response in venous blood ROS (increase at 15 days and subsequent decrease at 30 days) using comparable NLB and LED therapy. This discrepancy may reflect differences in our sampling protocol, which measured at days 0, 22, and 45, potentially missing the peak inflammatory phase that typically occurs within the first 21 days before transitioning to remodeling [76].

The absence of ROS fluctuations in the wound microenvironment in our study, despite clinical improvement, implies several possibilities: first, the treatments may act through pathways that bypass oxidative mechanisms; second, localized antioxidant effects may occur without detectable systemic changes; or third, our measurement intervals may have missed transient oxidative bursts. The stable ROS levels we observed at our selected timepoints likely represent measurements taken during the resolution phase of inflammation rather than indicating truly stable concentrations throughout treatment. This temporal consideration carries particular significance in diabetic wound healing, where prolonged persistence of inflammatory cells creates a sustained pro-inflammatory microenvironment characterized by excessive cytokines and ROS. Such conditions promote oxidative stress and significantly impair healing progression [77].

Although ROS levels were assessed at three key timepoints (D0, D22, and D45), this schedule may have missed transient oxidative bursts occurring during the early inflammatory phase (first 7–14 days). This limitation restricts the interpretation of ROS dynamics, particularly in the in situ wound microenvironment, where rapid fluctuations are expected. Notably, a reduction in systemic ROS levels was observed in venous blood at D22, suggesting a potential turning point in the systemic inflammatory response. However, this decrease does not fully capture the early local oxidative events, which may be critical to understanding the therapeutic mechanisms. Despite this temporal constraint, the EG2 group showed superior clinical outcomes, indicating that the curcumin-based therapy may exert localized anti-inflammatory or antioxidant effects beyond systemic modulation. These findings reinforce the need for future mechanistic studies with enhanced temporal resolution, especially during the acute inflammatory phase, to better characterize ROS behavior and therapeutic impact. These findings support the value of conducting mechanistic studies with refined temporal resolution to elucidate these effects better.

The curcumin-based formulation demonstrated significant therapeutic potential, evidenced by the stabilization of key inflammatory biomarkers (e.g., CRP, cytokines) in treated patients, suggesting localized anti-inflammatory effects despite systemic metabolic dysregulation. While ROS levels in the wound microenvironment remained stable throughout the study period, this absence of variation may reflect limitations in our sampling protocol, which potentially missed transient oxidative bursts during the critical early inflammatory phase (days 0–21) of wound healing. These findings underscore the importance of temporal alignment between therapeutic interventions and biological processes in diabetic wound repair. Future studies should prioritize high-frequency biomarker sampling during the initial healing stages to better elucidate the interplay between oxidative stress dynamics and therapeutic efficacy, while further validating the role of curcumin in modulating inflammatory pathways to overcome the chronicity of diabetic ulcers.

This study presents promising results for combinatorial therapy using a natural latex biomembrane with curcumin and LED phototherapy. However, limitations such as unequal ulcer severity between groups, restricted ROS sampling intervals, absence of statistical control for confounding factors, and the pilot-scale design must be acknowledged. These factors may influence the interpretation of the findings and should be addressed in future clinical trials with larger sample sizes and more rigorous stratification.

Methodological limitations, including the small sample size (*n* = 5/group), non-standardized control dressings, and baseline imbalances in wound severity (e.g., 40% Texas Grade 3 ulcers in CG), necessitate cautious interpretation. However, the clinically meaningful trends observed, particularly EG2’s superior performance over conventional therapy, emphasize the potential of targeted biomaterial–phototherapy combinations to address DFU complexity. Future studies should prioritize larger cohorts, standardized protocols, and temporal profiling of inflammatory/oxidative markers to optimize personalized treatment strategies and validate translational efficacy.

## 5. Study Limitations

This pilot study yielded promising preliminary results for the use of a natural latex biomembrane loaded with curcumin and combined with LED phototherapy in diabetic wound healing. However, some limitations must be acknowledged. The main concern was the imbalance in ulcer severity across groups. Due to ethical considerations under Brazilian guidelines (Resolution 466/2012), patients with more severe ulcers (Grade 3) were allocated to the control group. Although this introduces a potential confounder, it is noteworthy that the control group showed poor healing outcomes, while EG2 exhibited consistent wound closure. The limited sampling intervals for reactive oxygen species (ROS) quantification may have hindered a more precise understanding of the oxidative stress dynamics during treatment. No statistical differences were identified related to comorbidities or ulcer duration. Future trials should incorporate multivariable analyses and stratified randomization to minimize bias and improve internal validity. Despite these limitations, the pilot design was appropriate for the initial exploration of this combinatorial therapy. As in other early-phase trials, the findings provide important insights for future research design. Larger clinical studies with expanded treatment arms and more rigorous methodological controls are warranted to confirm the observed effects and better delineate the contribution of each component to the healing process.

## 6. Conclusions

This pilot study demonstrates that the combination of curcumin-loaded liposomes, natural latex biomembranes, and LED phototherapy (EG2) significantly enhances diabetic foot ulcer (DFU) healing, achieving an average of nearly 90% wound closure within 45 days, even in participants with poor glycemic control and long-standing ulcers. Although no significant intergroup differences in wound microenvironment ROS levels were observed, EG2 showed a notable systemic reduction in ROS concentrations on day 22, suggesting a transient yet relevant antioxidant effect during the mid-treatment phase.

These findings highlight the potential of smart biomaterial-based combinatorial strategies to partially overcome systemic metabolic challenges such as chronic hyperglycemia and oxidative stress. This study provides a strong foundation for advancing DFU treatment by integrating natural bioactives, nanotechnology, and phototherapy. Future research should focus on optimizing controlled-release formulations, synchronizing therapeutic timing with the biological phases of wound healing, and incorporating personalized tools to guide clinical decisions and improve patient outcomes.

## Data Availability

The raw data supporting the conclusions of this article will be made available by the authors on request.

## Supplementary Materials

The following supporting information can be downloaded at: https://www.mdpi.com/article/10.3390/pharmaceutics17060772/s1, Figure S1. Detection of reactive oxygen species in (A, B, C) venous blood, (D, E, F) wound blood, and (G, H, I) wound tissues in participants who used the SUS gold standard therapy (CG), natural latex biomembrane in association with LED irradiation (EG1), and natural latex biomembrane containing liposomes with curcumin in association with LED irradiation (EG2) on days 0, 22, and 45. Table S1: Tests, reference values, and methods used for complete blood count and biochemical assays in patients with diabetic foot ulcers; Table S2: Demographic and clinical characteristics of the studied population undergoing the interventions: protocol used in the Brazilian Unified Health System—BUHS—(CG), or experimental protocols using latex-based dressing and red LED (EG1) or latex containing curcumin and red LED (EG2); Table S3: Characterization of the study population according to the intervention groups: BUHS protocol (CG), latex-based dressing with red LED (EG1), and latex containing curcumin with red LED (EG2); Table S4: Classification of wounds in the study population by type and grade according to the University of Texas Diabetic Wound Classification; Table S5:. Hematological parameters of diabetic patients with foot ulcers at baseline (Day 0) undergoing treatment with the BUHS protocol, NLB + red LED, and NLB with curcumin + red LED; Table S6: Hematological test results of diabetic patients with foot ulcers who used the BUHS protocol for diabetic ulcer treatment; Table S7: Hematological test results of diabetic patients with foot ulcers treated with latex and red LED; Table S8: Results of hematological examinations of diabetics with foot ulcers who used latex containing curcumin and red LED for the treatment of diabetic ulcers; Table S9: Results of biochemical tests of diabetic patients with foot ulcers who used the BUHS protocol, latex and red LED, and latex containing curcumin and red LED for the treatment of diabetic ulcers on day 0 of the protocols; Table S10: Results of biochemical tests of diabetics with foot ulcers who used the BUHS protocol for the treatment of diabetic ulcers; Table S11: Results of biochemical tests of diabetics with foot ulcers who used latex and red LED for the treatment of diabetic ulcers; Table S12: Results of biochemical tests of diabetics with foot ulcers who used latex containing curcumin and red LED for the treatment of diabetic ulcers. References [37,78,79] are cited in the Supplementary Materials.

## Abbreviations

The following abbreviations are used in this manuscript:

DFU	Diabetic foot ulcers
WIfI	Wound, ischemia, and foot infection
MMP	Matrix metalloproteinases
ROS	Reactive oxygen species
AGEs	Advanced glycation end products
NLB	Natural latex biomembrane
PBM	Photobiomodulation
LED	Light-emitting diode
ATP	Adenosine triphosphate
CG	Control group
EG1	Experimental group 1
EG2	Experimental group 2
BMI	Body mass index
HbA1c	Glycated hemoglobin
ROI	Regions of interest
EPR	Electron paramagnetic resonance
CMH	1-hydroxy-3-methoxycarbonyl-2,2,5,5-tetramethylpyrrolidine
KHB	Krebs HEPES buffer
DETC	Sodium diethyldithiocarbamate trihydrate
CP^•^	3-carboxy-proxyl nitroxide
CM^•^	3-methoxycarbonyl-2,2,5,5-tetramethylpyrrolidine-1-oxyl
DM	Diabetes mellitus
MCV	Mean corpuscular volume
MCH	Mean corpuscular hemoglobin
MCHC	Mean corpuscular hemoglobin concentration
RDW	Red cell distribution width
MPV	Mean platelet volume
CRP	C-reactive protein
AST	Aspartate Aminotransferase
ALT	Alanine Aminotransferase
WCP	Wound closure percentage
BUHS	Brazilian Unified Health System
FBN-1	Fibrillin-1
SBD	Brazilian diabetes society
SD	Standard deviation
SBACV	Brazilian Society of Angiology and Vascular Surgery
