# Supplementary material for: A Natural Latex-Based Smart Dressing for Curcumin Delivery Combined with LED Phototherapy in Diabetic Foot Ulcers: A Pilot Clinical Study
Source: Pharmaceutics. 2025 Jun 12;17(6):772. doi: 10.3390/pharmaceutics17060772 (PMC12196574; doi:10.3390/pharmaceutics17060772)
Supplement: Supplementary file 1 [file pharmaceutics-17-00772-s001.zip › pharmaceutics-3648003-supplementary.pdf]

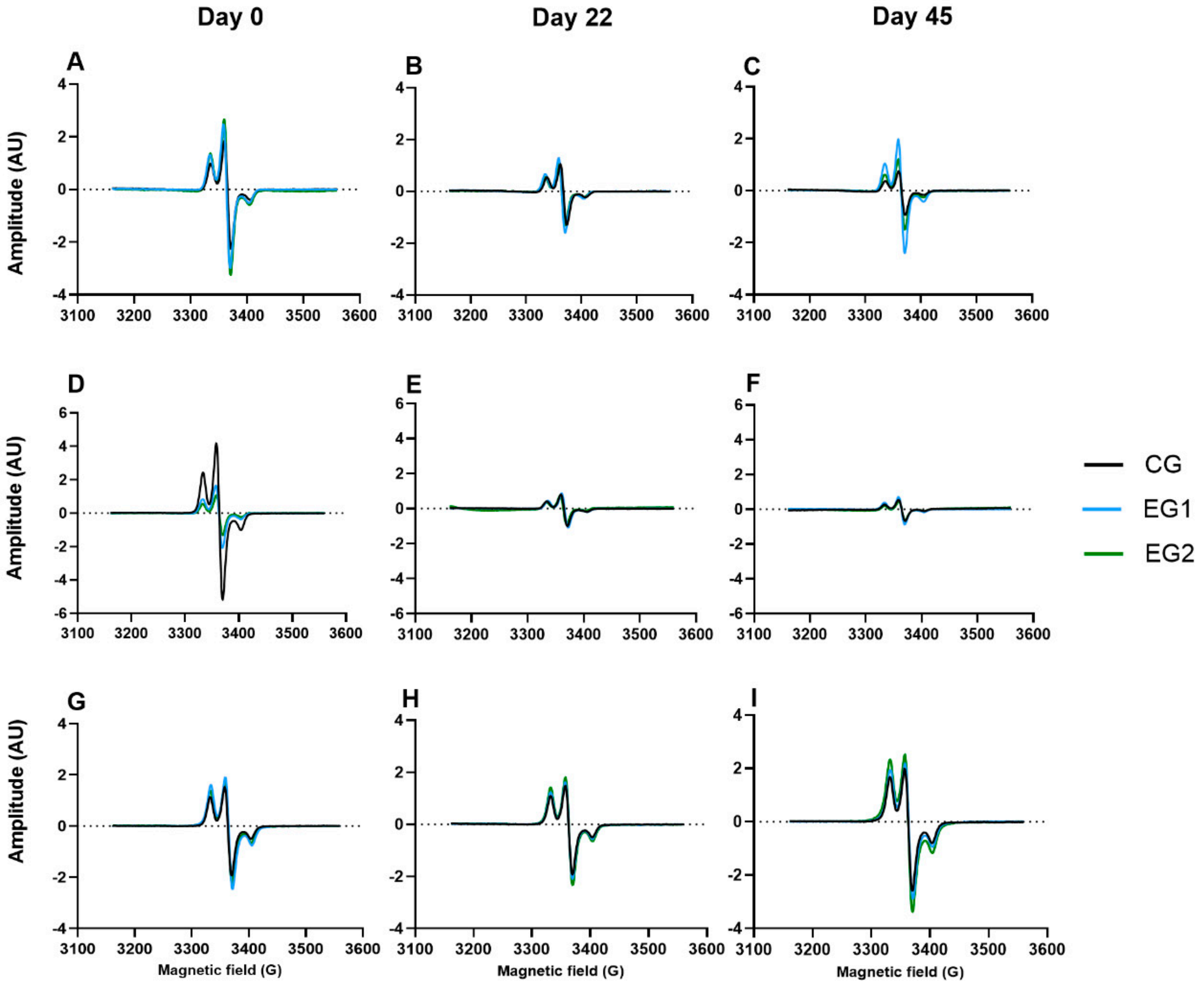

Figure S1. Detection of reactive oxygen species in **(A, B, C)** venous blood, **(D, E, F)** wound blood and **(G, H, I)** wound tissues in participants who used the SUS gold standard therapy (CG), natural latex biomembrane in association with LED irradiation (EG1) and natural latex biomembrane containing liposomes with curcumin in association with LED irradiation (EG2) on days 0, 22 and 45. Representative spectra of CM radical detected at 77 K.

Table S1. Tests, reference values, and methods used for complete blood count and biochemical assays in patients with diabetic foot ulcers. Sources: [37]; [a] Reference values provided by the Sabin Institute.

Test	Reference Values	Method
Red Blood Cells	3.9 to 5.8 million/mm^3	Fluorescent flow cytometry and impedance
Hemoglobin	11.5 to 16.9 g/dL	Fluorescent flow cytometry and impedance
Hematocrit	35.3 to 52.0%	Fluorescent flow cytometry and impedance
Mean Corpuscular Volume (MCV)	81.0 to 100.6 fL	Fluorescent flow cytometry and impedance
Mean Corpuscular Hemoglobin (MCH)	26.3 to 32.6 pg	Fluorescent flow cytometry and impedance
Mean Corpuscular Hemoglobin Concentration (MCHC)	30.5 to 34.6 g/dL	Fluorescent flow cytometry and impedance
Red Cell Distribution Width (RDW)	11.9 to 15.5%	Fluorescent flow cytometry and impedance
White Blood Cells	3600 to 11000 /mm^3	Fluorescent flow cytometry and impedance
Segmented neutrophils	1480 to 7700 /mm^3	Fluorescent flow cytometry and impedance
Eosinophils	0 to 550 /mm^3	Fluorescent flow cytometry and impedance
Basophils	0 to 220 /mm^3	Fluorescent flow cytometry and impedance
Lymphocytes	740 to 5500 /mm^3	Fluorescent flow cytometry and impedance
Monocytes	37 to 1500 /mm^3	Fluorescent flow cytometry and impedance
Plaquetas	130 to 450 x10^3/mm^3	Fluorescent flow cytometry and impedance
Mean Platelet Volume (MPV)	6.8 to 12.6 fL [a]	Fluorescent flow cytometry and impedance
Glucose	≥ 126 mg/dL [a]	Hexokinase
Glycated Hemoglobin - HbA1c	> 6.4% [a]	Turbidimetric inhibition immunoassay
Ultrasensitive PCR	< 0.5 mg/dL [a]	Latex-enhanced immunoturbidimetry
Urea	19 to 49 mg/dL [a]	Urease with GLDH
Creatinine	0.53 to 1.20 mg/dL [a]	amidinohydrolase /Oxidase
Serum aspartate aminotransferase (AST)	< 34 U/L [a]	IFCC without pyridoxal phosphate
Serum alanine aminotransferase (ALT)	10 to 49 U/L [a]	IFCC without pyridoxal phosphate
Alkaline Phosphatase	46 to 116 U/L [a]	Kinetic colorimetric
Total and Fractionated Bilirubin	0 to 1.20 mg/dL [a]	Vanadate with oxidation
Albumin	3.2 to 4.8 g/dL [a]	Bromocresol Green
Cortisol	5.27 to 22.45 µg/dL [a]	Chemiluminescence

Table S2. Demographic and clinical characteristics of the studied population undergoing the interventions: protocol used in the Brazilian Unified Health System – BUHS – (CG), or experimental protocols using latex-based dressing and red LED (EG1), or latex containing curcumin and red LED (EG2).

**Variable**		**CG – BUHS Protocol (%)**	**EG1 – NLB + LED (%)**	**EG2 – NLB with Curcumin + LED (%)**	**All Participants (%)**
Sex	Female	20%	20%	20%	20%
	Male	80%	80%	80%	80%
Marital status	Single	20%	20%	0%	13%
	Married	60%	60%	80%	67%
	Divorced	20%	20%	0%	13%
	Widowed	0%	0%	20%	7%
Current employment status	Employed	40%	40%	60%	47%
	Unemployed	40%	40%	0%	27%
	Retired	20%	20%	40%	27%
Hypertension	Yes	60%	80%	60%	67%
	No	40%	20%	40%	33%
Type of Treatment	Insulin	40%	80%	60%	60%
	Oral	60%	20%	40%	40%
Obesity	Yes	40%	40%	20%	33%
	No	60%	60%	80%	67%
Mobility	Independent	40%	60%	40%	47%
	Partially dependent	40%	20%	40%	33%
	Dependent	20%	20%	20%	20%
Smoker	Yes	0%	0%	20%	7%
	No	100%	100%	80%	93%
Alcoholic	Yes	40%	20%	40%	33%
	No	60%	80%	60%	67%
Participants (n)		5	5	5	15

Table S3. Characterization of the study population according to the intervention groups: BUHS protocol (CG), latex-based dressing with red LED (EG1), and latex containing curcumin with red LED (EG2).

	**CG – BUHS Protocol**			**EG1 – NLB + LED**			**EG2 – NLB with Curcumin + LED**			**All Participants**		
Variable	Mean ± SD	Median	CV	Mean ± DP	Median	CV	Mean ± DP	Median	CV	Mean ± DP	Median	CV
Age (years)	57±9.92	59	17%	56±9.92	57	18%	63±9.92	60	16%	59±13.36	59	23%
Weight (kg)	84.94±17.90	75	21%	91±18.18	82	20%	74.24±16.74	75	23%	83.39±17.82	81.4	21%
Height (m)	1.71±0.10	1.72	6%	1.74±0.12	1.68	7%	1.68±0.10	1.66	6%	1.71±0.10	1.69	6%
BMI (kg/m²)	29±5.60	27	19%	30±2.43	30	8%	26±6.52	25	25%	28±5.00	27	18%
Duration of DM (years)	12±9.09	13	77%	15±6.53	15	44%	9±7.64	7	89%	12±7.70	13	66%
Ulcer duration (months)	22±23.94	6	110%	33±49.30	12	149%	7±9.48	4	128%	21±31.64	6	153%
HbA1C (%)	7.8±1.74	8	22%	6.8±0.15	6.8	2%	8.7±2.72	7	31%	7.9±2.02	6.9	26%
Glucose (mg/dL)	226±158.29	186	70%	137±46.16	153	33%	137±34.19	146	25%	170±102.35	151	60%

Note: SD: standard deviation, CV: coefficient of variation. Kruskal-Wallis test followed by Dunn’s post hoc test.

Table S4. Classification of wounds in the study population by type and grade according to the University of Texas Diabetic Wound Classification.

		CG – BUHS Protocol (%)	EG1 – NLB + LED (%)	EG2 – Latex with Curcumin + LED (%)	All Participants (%)
Type of Wound	Neuropathic [a]	100%	60%	40%	67%
	Venous [b]	0%	20%	20%	13%
	Traumatic [c]	0%	0%	20%	7%
	Neuroischemic [d]	0%	20%	20%	13%
Grade (Texas Classification)	0	0%	0%	0%	0%
	1	60%	80%	60%	67%
	2	0%	20%	40%	20%
	3	40%	0%	0%	13%

Notes: [a]Ulcer caused by loss of protective sensation due to peripheral neuropathy; [b] Ulcer caused by venous stasis; [c] Ulcer resulting from trauma. such as penetrating injury or high/repetitive pressure; [d] Ulcer with neuropathic features and vascular compromise (ischemia). [78,79]

Table S5. Hematological parameters of diabetic patients with foot ulcers at baseline (Day 0) undergoing treatment with the BUHS protocol, latex + red LED, and latex with curcumin + red LED.

DAY 0									
	**CG – BUHS Protocol**			**EG1 – NLB + LED**			**EG2 – NLB with Curcumin + LED**		
**Parameter**	**Mean ± SD**	**Median**	**CV**	**Mean ± SD**	**Median**	**CV**	**Mean ± SD**	**Median**	**CV**
Red blood cells (×10⁶/mm³)	4.9±0.5	4.82	10%	4.48±0.6	4.41	14%	4.62±0.7	4.78	15%
Hemoglobin (g/dL)	13.68±2.1	14.4	15%	12.7±1.4	12.8	11%	13.28±1.9	13.7	14%
Hematocrit (%)	40.9±5.1	39.8	12%	38.33±5.1	39.0	13%	39.82±6.5	40.3	16%
MCV (fl)	83.28±2.8	83.6	3%	85.6±2.4	84.4	3%	86.34±5.3	86.7	6%
MCH (pg)	27.82±2.2	28.6	8%	28.43±1.0	29.0	3%	28.88±1.6	29.0	6%
MCHC (g/dL)	33.36±1.9	33.1	6%	33.2±1.0	32.8	3%	33.48±1.1	33.3	3%
RDW (%)	13.38±1.3	12.6	10%	13.7±1.3	14.2	10%	13.64±1.2	13.7	9%
Leukocytes (mm³)	8660.0±1738.7	8.300.0	20%	9200.0±1479.9	8.500.0	16%	6840.0±1128.3	6.400.0	16%
Segmented neutrophils (mm³)	5381.0±1671.2	5.146.0	31%	5539.0±1086.2	5.185.0	20%	3614.0±1069.8	3.256.0	30%
Eosinophils (mm³)	410.4±339.0	325.0	83%	477.0±247.8	340.0	52%	182.0±119.8	192.0	66%
Basophils (mm³)	67.2±41.0	78.0	61%	64.67±57.3	85.0	89%	57.2±33.2	64.0	58%
Lymphocytes (mm³)	2239.2±554.7	2.418.0	25%	2502.67±205.5	2.507.0	8%	2341.4±638.6	2.125.0	27%
Monocytes (mm³)	562.2±72.4	581.0	13%	616.67±136.8	595.0	22%	645.4±241.5	595.0	37%
Platelets (x10³/mm³)	332.8±174.2	331.0	52%	289.0±97.9	252.0	34%	299.6±58.6	272.0	20%
Mean platelet volume – MPV (fL)	11.04±1.2	11.0	11%	11.0±1.5	10.7	13%	10.82±0.7	11.0	7%

Note: SD: standard deviation, CV: coefficient of variation. Kruskal-Wallis test followed by Dunn's post hoc test.

Table S6. Hematological test results of diabetic patients with foot ulcers who used the BUHS protocol for diabetic ulcer treatment.

GC – BUHS PROTOCOL									
	D0			D22			D45		
**Parameters**	**Mean ± SD**	**Median**	**CV**	**Mean ± SD**	**Median**	**CV**	**Mean ± SD**	**Median**	**CV**
Red blood cells (×10⁶/mm³)	4.9±0.5	4.82	10%	4.9±0.6	5.0	12%	5.3±0.3	5.1	6%
Hemoglobin (g/dL)	13.68±2.1	14.4	15%	13.4±1.9	14.2	14%	14.0±0.7	14.0	5%
Hematocrit (%)	40.9±5.1	39.8	12%	40.0±5.1	41.6	13%	42.4±2.4	42.0	6%
MCV (fl)	83.28±2.8	83.6	3%	81.1±3.7	80.9	5%	80.8±7.3	78.9	9%
MCH (pg)	27.82±2.2	28.6	8%	27.1±1.7	26.3	6%	26.7±0.9	26.2	3%
MCHC (g/dL)	33.36±1.9	33.1	6%	33.4±1.3	32.7	4%	33.1±2.0	33.3	6%
Red blood cells (×10⁶/mm³)	13.38±1.3	12.6	10%	13.6±1.4	13.4	10%	13.5±0.4	13.7	3%
Leukocytes (mm³)	8660.0±1738.7	8.300.0	20%	8280.0±2572.4	7.500.0	31%	7500.0±854.4	7.400.0	11%
Segmented neutrophils (mm³)	5381.0±1671.2	5.146.0	31%	4937.0±2751.3	3.975.0	56%	3748.0±1129.5	3.256.0	30%
Eosinophils (mm³)	410.4±339.0	325.0	83%	444.6±501.0	288.0	113%	189.0±130.4	148.0	69%
Basophils (mm³)	67.2±41.0	78.0	61%	82.8±25.7	75.0	31%	75±8.5	74.0	11%
Lymphocytes (mm³)	2239.2±554.7	2.418.0	25%	2234.6±735.9	2.232.0	33%	2773.0±546.9	2.479.0	20%
Monocytes (mm³)	562.2±72.4	581.0	13%	581.0±141.1	558.0	24%	715.0±180.0	756.0	25%
Platelets (x10³/mm³)	332.8±174.2	331.0	52%	330.6±179.2	383.0	54%	336.7±130.3	398.0	39%
Mean platelet volume - MPV (fL)	11.04±1.2	11.0	11%	11.1±1.2	11.9	10%	10.4±0.5	10.5	5%

Note: Treatment moments: D0: day 0, D22: day 22, and D45: day 45. SD: standard deviation, CV: coefficient of variation. Friedman test followed by Dunn's post hoc test.

Table S7. Hematological test results of diabetic patients with foot ulcers treated with latex and red LED

EG1 – NLB + LED									
	D0			D22			D45		
Parameters	**Mean ± SD**	**Median**	**CV**	**Mean ± SD**	**Median**	**CV**	**Mean ± SD**	**Median**	**CV**
Red Blood Cells (million/mm³)	4.48±0.6	4.41	14%	4.5±0.5	4.3	11%	4.8±0.6	4.5	13%
Hemoglobin (g/dL)	12.7±1.4	12.8	11%	12.9±1.1	12.4	9%	13.9±1.9	13.6	14%
Hematocrit (%)	38.33±5.1	39.0	13%	37.6±3.5	36.9	9%	41.1±4.6	40.5	11%
MCV (fL)	85.6±2.4	84.4	3%	84.4±2.2	84.6	3%	86±3.4	87.0	4%
MCH (pg)	28.43±1.0	29.0	3%	28.9±0.7	29.0	2%	29.0±1.6	28.9	5%
MCHC (g/dL)	33.2±1.0	32.8	3%	34.3±0.7	34.3	2%	33.7±1.2	33.4	3%
RDW (%)	13.7±1.3	14.2	10%	13.4±0.9	13.8	7%	13.1±1.0	12.9	8%
Leukocytes (mm³)	9200.0±1479.9	8.500.0	16%	11066.7±3350.1	10400.0	30%	9840.0±3159.6	10400.0	32%
Segmented neutrophils (mm³)	5539.0±1086.2	5.185.0	20%	6781.3±2175.1	6.760.0	32%	6207.6±2457.4	6.344.0	40%
Eosinophils (mm³)	477.0±247.8	340.0	52%	616.7±357.1	416.0	58%	387.2±171.4	312.0	44%
Basophils (mm³)	64.67±57.3	85.0	89%	83.7±75.6	104.0	90%	80.6±54.8	104.0	68%
Lymphocytes (mm³)	2502.67±205.5	2.507.0	8%	2837.3±606.4	2.592.0	21%	2411.0±598.6	2.684.0	25%
Monocytes (mm³)	616.67±136.8	595.0	22%	698.7±199.6	728.0	29%	753.6±218.3	832.0	29%
Platelets (x10³/mm³)	289.0±97.9	252.0	34%	301.7±141.5	224.0	47%	271.4±83.9	295.0	31%
Mean Platelet Volume – MPV (fL)	11.0±1.5	10.7	13%	11.0±1.5	10.3	14%	11.0±1.6	10.2	15%

Note: Treatment time points: D0: day 0, D22: day 22, and D45: day 45. SD: standard deviation, CV: coefficient of variation. Friedman test followed by Dunn's post hoc test.

Table S8. Results of hematological examinations of diabetics with foot ulcers who used latex containing curcumin and red LED for the treatment of diabetic ulcers.

EG2 – LATEX CONTAINING CURCUMIN + LED									
	**D0**			**D22**			**D45**		
Parameters	**Mean ± SD**	**Median**	**CV**	**Mean ± SD**	**Median**	**CV**	**Mean ± SD**	**Median**	**CV**
Red Blood Cells (million/mm³)	4.62±0.7	4.78	15%	4.4±0.9	4.5	21%	4.5±0.9	5.0	21%
Hemoglobin (g/dL)	13.28±1.9	13.7	14%	12.6±2.2	13.5	17%	13.3±2.5	14.6	18%
Hematocrit (%)	39.82±6.5	40.3	16%	37.6±6.2	41.1	17%	40.3±7.6	43.0	19%
MCV (fL)	86.34±5.3	86.7	6%	85.5±7.2	85.5	8%	89.9±3.6	90.6	4%
MCH (pg)	28.88±1.6	29.0	6%	28.7±1.8	28.6	6%	29.8±0.9	29.3	3%
MCHC (g/dL)	33.48±1.1	33.3	3%	33.6±1.2	33.2	4%	33.1±0.9	33.1	3%
RDW (%)	13.64±1.2	13.7	9%	13.8±1.2	13.7	8%	14.2±1.1	14.4	8%
Leukocytes (mm³)	6840.0±1128.3	6.400.0	16%	7100.0±1407.1	7.600.0	20%	6800.0±692.8	6.400.0	10%
Segmented neutrophils (mm³)	3614±1069.8	3.256.0	30%	4085.0±1093.6	4.576.0	27%	3644.0±992.8	3.136.0	27%
Eosinophils (mm³)	182.0±119.8	192.0	66%	164.4±110.0	176.0	67%	212.0±145.5	128.0	69%
Basophils (mm³)	57.2±33.2	64.0	58%	45.4±42.5	62.0	94%	68.0±6.9	64.0	10%
Lymphocytes (mm³)	2341.4±638.6	2.125.0	27%	2192.0±824.3	1.860.0	38%	2016.0±178.2	2.048.0	9%
Monocytes (mm³)	645.4±241.5	595.0	37%	613.2±316.7	558.0	52%	860.0±284.1	1.024.0	33%
Platelets (x10³/mm³)	299.6±58.6	272.0	20%	248.0±86.5	215.0	35%	264±77.2	224.0	29%
Mean Platelet Volume – MPV (fL)	10.82±0.7	11.0	7%	11.2±0.6	11.2	5%	11.5±0.5	11.3	5%

Note: Treatment moments: D0: day 0, D22: day 22, and D45: day 45. SD: standard deviation. CV: coefficient of variation. Friedman test followed by Dunn's post hoc test.

Table S9. Results of biochemical tests of diabetic patients with foot ulcers who used the BUHS protocol, latex and red LED, and latex containing curcumin and red LED for the treatment of diabetic ulcers on day 0 of the protocols.

DAY 0									
	CG – BUHS Protocol			EG1 – NLB + LED			EG2 – Latex with Curcumin + LED		
**Parameters**	**Mean ± SD**	**Median**	**CV**	**Mean ± SD**	**Median**	**CV**	**Mean ± SD**	**Median**	**CV**
Glucose (mg/dL)	226.4±158.3	186	70%	137±56.0	156	41%	137±34.2	146	25%
Glycated hemoglobin – HbA1c (%)	7.76±1.7	8	22%	6.77±0.2	6.8	2%	8.68±2.7	7	31%
Ultrasensitive PCR (mg/dL)	1.51±0.9	1.32	57%	0.95±1.2	0.46	124%	1.36±2.0	0.64	144%
Urea (mg/dL)	49.6±10.8	49	22%	50.67±15.1	44	30%	54.6±56.2	33	103%
Creatinine (mg/dL)	0.85±0.2	0.83	22%	1.18±0.5	1.06	38%	1.01±0.7	0.79	68%
Aspartate aminotransferase (AST)	40.8±11.1	41	27%	19.00±3.5	17	18%	51.4±40.7	32	79%
Alanine aminotransferase (ALT)	38.6±6.8	37	18%	17.67±4.2	19	24%	57.2±45.6	43	80%
Alkaline phosphatase (U/L)	111.8±52.7	81	47%	80.33±44.1	59	55%	104±30.4	99	29%
Total bilirubin (mg/dL)	0.42±0.2	0.32	54%	0.28±0.2	0.26	73%	0.41±0.1	0.38	34%
Direct bilirubin (mg/dL)	0.13±0.1	0.12	42%	0.09±0.1	0.08	55%	0.14±0	0.14	26%
Indirect bilirubin (mg/dL)	0.29±0.2	0.21	62%	0.19±0.2	0.18	82%	0.28±0.1	0.25	43%
Albumin (g/dL)	4.4±0.6	4	15%	4.4±0.2	4.4	5%	4.06±0.3	4.2	7%
Cortisol (µg/dL)	15.65±6.0	11.95	38%	8.77±5.0	8.96	57%	11.75±3.4	10.95	29%

Note: SD: standard deviation. CV: coefficient of variation. Kruskal-Wallis test followed by Dunn's post hoc test.

Table S10. Results of biochemical tests of diabetics with foot ulcers who used the BUHS protocol for the treatment of diabetic ulcers.

CG – BUHS PROTOCOL									
	D0			D22			D45		
**Parameters**	**Mean ± SD**	**Median**	**CV**	**Mean ± SD**	**Median**	**CV**	**Mean ± SD**	**Median**	**CV**
Glucose (mg/dL)	226.4±158.3	186	70%	**284.2±162.6**[a]	278	57%	274.0±103.0	272	38%
Glycated hemoglobin – HbA1c (%)	7.76±1.7	8	22%	9.0±3.6	7.6	40%	9.1±3.8	7.3	42%
Ultrasensitive PCR (mg/dL)	1.51±0.9	1.32	57%	2.3±3.3	1.1	143%	2.3±2.6	0.9	116%
Urea (mg/dL)	49.6±10.8	49	22%	50.4±16.7	55	33%	45.7±19.1	53	42%
Creatinine (mg/dL)	0.85±0.2	0.83	22%	0.9±0.2	1	21%	0.9±0.3	0.9	36%
Aspartate aminotransferase (AST)	40.8±11.1	41	27%	37.0±26.4	31	71%	48.7±25.3	44	52%
Alanine aminotransferase (ALT)	38.6±6.8	37	18%	48.2±46.1	33	96%	62.7±52.0	46	83%
Alkaline phosphatase (U/L)	111.8±52.7	81	47%	107.0±54.7	81.5	51%	110.0±39.1	91	36%
Total bilirubin (mg/dL)	0.42±0.2	0.32	54%	0.4±0.3	0.3	61%	0.5±0.1	0.5	23%
Direct bilirubin (mg/dL)	0.13±0.1	0.12	42%	0.1±0.1	0.1	56%	0.1±0	0.1	8%
Indirect bilirubin (mg/dL)	0.29±0.2	0.21	62%	0.3±0.2	0.2	66%	0.4±0.1	0.4	29%
Albumin (g/dL)	4.4±0.6	4	15%	4.2±0.4	4.1	9%	4.4±0.3	4.2	8%
Cortisol (µg/dL)	15.65±6.0	11.95	38%	15.8±5.3	13.9	33%	12.8±5.1	10.8	40%

Note: Treatment moments: D0: day 0, D22: day 22, and D45: day 45. SD: standard deviation. CV: coefficient of variation. Friedman test followed by Dunn's post hoc test. [a] p-value=0.043 when compared to D0.

Table S11. Results of biochemical tests of diabetics with foot ulcers who used latex and red LED for the treatment of diabetic ulcers.

EG1 – NLB + LED									
	D0			D22			D45		
**Parameters**	**Mean ± SD**	**Median**	**CV**	**Mean ± SD**	**Median**	**CV**	**Mean ± SD**	**Median**	**CV**
Glucose (mg/dL)	137.0±56.0	156	41%	150.0±63.6	155	42%	150.4±58.3	143	39%
Glycated hemoglobin – HbA1c (%)	6.77±0.2	6.8	2%	6.9±0.4	7	6%	7.6±0.8	7.5	10%
Ultrasensitive RCP (mg/dL)	0.95±1.2	0.46	124%	1.3±1.2	1.2	95%	1.2±1.4	0.7	113%
Urea (mg/dL)	50.67±15.1	44	30%	63.7±24.6	72	39%	52.4±18.3	45	35%
Creatinine (mg/dL)	1.18±0.5	1.06	38%	1.4±0.6	1.5	42%	1.0±0.3	1	31%
Aspartate aminotransferase (AST)	19.0±3.5	17	18%	18.0±2.6	17	15%	41.8±36.7	27	88%
Alanine aminotransferase (ALT)	17.67±4.2	19	24%	19±2.0	19	11%	40.4±25.5	33	63%
Alkaline phosphatase (U/L)	80.3±44.1	59	55%	88±48.4	69	55%	104.4±42.4	88	41%
Total bilirubin (mg/dL)	0.28±0.2	0.26	73%	0.3±0.2	0.4	50%	0.5±0.2	0.4	45%
Direct bilirubin (mg/dL)	0.09±0.1	0.08	55%	0.1±0.1	0.1	47%	0.2±0.1	0.1	64%
Indirect bilirubin (mg/dL)	0.19±0.2	0.18	82%	0.2±0.1	0.2	54%	0.3±0.1	0.3	38%
Albumin (g/dL)	4.4±0.2	4.4	5%	4.5±0.1	4.5	2%	4.5±0.4	4.4	8%
Cortisol (µg/dL)	8.77±5.0	8.96	57%	9.2±1.7	9.8	19%	11.4±2.5	12	22%

Note: Treatment moments: D0: day 0, D22: day 22, and D45: day 45. SD: standard deviation. CV: coefficient of variation. Friedman test followed by Dunn's post hoc test.

Table S12. Results of biochemical tests of diabetics with foot ulcers who used latex containing curcumin and red LED for the treatment of diabetic ulcers.

EG2 – NLB WITH CURCUMIN + LED									
	D0			D22			D45		
**Parameters**	**Mean ± SD**	**Median**	**CV**	**Mean ± SD**	**Median**	**CV**	**Mean ± SD**	**Median**	**CV**
Glucose (mg/dL)	137.0±34.2	146	25%	138.0±43.4	155	31%	121.0±37.4	103	31%
Glycated hemoglobin – HbA1c (%)	8.68±2.7	7	31%	8.1±2.0	7	24%	**7.8±2.6**[a]	6.4	33%
Ultrasensitive PCR (mg/dL)	1.36±2.0	0.64	144%	1.2±1.6	0.2	135%	2.4±2.1	3.5	86%
Urea (mg/dL)	54.6±56.2	33	103%	47.0±45.2	29	96%	58.3±50.5	42	87%
Creatinine (mg/dL)	1.01±0.7	0.79	68%	1.0±0.6	0.8	64%	1.1±0.8	0.8	67%
Aspartate aminotransferase (AST)	51.4±40.7	32	79%	31.2±16.9	25	54%	29.0±11.3	23	39%
Alanine aminotransferase (ALT)	57.2±45.6	43	80%	34.8±24.4	25	70%	25.7±9.3	30	36%
Alkaline phosphatase (U/L)	104.0±30.4	99	29%	97.2±12.5	97	13%	96.0±6.9	92	7%
Total bilirubin (mg/dL)	0.41±0.1	0.38	34%	0.5±0.2	0.5	38%	0.7±0.6	0.4	87%
Direct bilirubin (mg/dL)	0.14±0	0.14	26%	0.2±0.1	0.2	34%	0.2±0.2	0.1	72%
Indirect bilirubin (mg/dL)	0.28±0.1	0.25	43%	0.4±0.2	0.4	43%	0.5±0.5	0.3	94%
Albumin (g/dL)	4.06±0.3	4.2	7%	4.1±0.4	4.1	10%	4.2±0.2	4.2	5%
Cortisol (µg/dL)	11.75±3.4	10.95	29%	11.5±5.1	11.4	44%	11.5±1.4	10.8	12%

Note: Treatment moments: D0: day 0, D22: day 22, and D45: day 45. SD: standard deviation. CV: coefficient of variation. Friedman test followed by Dunn's post hoc test. [a] p-value=0.043 when compared to D0.
